# Ampere-level current density ammonia electrochemical synthesis using CuCo nanosheets simulating nitrite reductase bifunctional nature

**DOI:** 10.1038/s41467-022-35533-6

**Published:** 2022-12-22

**Authors:** Jia-Yi Fang, Qi-Zheng Zheng, Yao-Yin Lou, Kuang-Min Zhao, Sheng-Nan Hu, Guang Li, Ouardia Akdim, Xiao-Yang Huang, Shi-Gang Sun

**Affiliations:** 1 State Key Laboratory of Physical Chemistry of Solid Surfaces, Department of Chemistry, College of Chemistry and Chemical Engineering, Xiamen University, Xiamen, 361005 China; 2 School of Chemical and Environmental Engineering, College of Chemistry, Chemical Engineering and Materials Science, Soochow University, Suzhou, Jiangsu 215123 China; 3 Cardiff Catalysis Institute, School of Chemistry, Cardiff University, Main Building, Park Place, Cardiff, CF10 3AT UK

**Keywords:** Electrocatalysis, Pollution remediation, Electrocatalysis

## Abstract

The development of electrocatalysts capable of efficient reduction of nitrate (NO_3_^−^) to ammonia (NH_3_) is drawing increasing interest for the sake of low carbon emission and environmental protection. Herein, we present a CuCo bimetallic catalyst able to imitate the bifunctional nature of copper-type nitrite reductase, which could easily remove NO_2_^−^ via the collaboration of two active centers. Indeed, Co acts as an electron/proton donating center, while Cu facilitates NO_x_^−^ adsorption/association. The bio-inspired CuCo nanosheet electrocatalyst delivers a 100 ± 1% Faradaic efficiency at an ampere-level current density of 1035 mA cm^−2^ at −0.2 V *vs*. Reversible Hydrogen Electrode. The NH_3_ production rate reaches a high activity of 4.8 mmol cm^−2^ h^−1^ (960 mmol g_cat_^−1^ h^−1^). A mechanistic study, using electrochemical in situ Fourier transform infrared spectroscopy and shell-isolated nanoparticle enhanced Raman spectroscopy, reveals a strong synergy between Cu and Co, with Co sites promoting the hydrogenation of NO_3_^−^ to NH_3_ via adsorbed *H species. The well-modulated coverage of adsorbed *H and *NO_3_ led simultaneously to high NH_3_ selectivity and yield.

## Abstract (web summary)

Electroreduction of NO_3_^−^ to NH_3_ is drawing increasing interest. Here, the authors designed a CuCo catalyst imitating the bifunctional nature of Cu-type nitrite reductase to deliver an ampere-level current density for NH_3_ formation.

## Introduction

Nitrate anions (NO_3_^−^), widely present in industrial and agricultural wastewater, pose a real potential threat to human health and ecological balance, especially their incomplete conversion into nitrites (NO_2_^−^) which are thought to be cancerogenic by inducing liver damage and methaemoglobinaemia^1^. The conventional biological treatments for NO_3_^−^ removal into nitrogen (N_2_) gas, involving nitrification and denitrification processes, are energy intensive (~11.7 to 12.5 kWh kg N^−1^)^2^. Actually, the reduction of NO_3_^−^ into NH_3_ has become of great interest from an industrial point of view since NH_3_ is a highly important industrial chemical for the synthesis of pharmaceuticals, fertilizers, dyes, plastic, etc.^3^, and is also considered for hydrogen storage/release as a carbon-free hydrogen carrier^4^. To date, the industrial synthesis of NH_3_ relies heavily on the non-sustainable and eco-unfriendly Haber–Bosch route, which requires harsh conditions i.e., high temperature (400–600 °C) and high pressure (200–350 atm), and heavily relies on fossil energy^3^. The total amount of CO_2_ produced during the Haber–Bosch process accounts for roughly 1.2% of the global annual CO_2_ emissions, more than any other industrial chemicals synthesis^5^.

Electrocatalytic reduction of NO_3_^−^ into NH_3_, powered by green energy, has drawn increasing attention and is considered as a sustainable complementary process to the Haber–Bosch process^2,6,7^, since this technology can simultaneously meet the 21st session of the Conference of the Parties (COP) two-degree scenario (2DS) target for NH_3_ and protect the environment from the eutrophic water pollution. The rational design of novel electrocatalysts with both high activity and selectivity is crucial for reducing NO_3_^−^ (NO_3_^−^RR) and achieving large-scale applications, and satisfying high industrial demands. Since Nature has developed sophisticated and efficient machinery such as enzymes^8,9^, it is interesting to design catalysts by studying the mechanism of enzymatic reduction. Indeed, biocatalytic reduction of NO_3_^−^ to NH_3_ widely exists inside many microorganisms, where NO_3_^−^ ions are firstly reduced into NO_2_^−^ by NO_3_^−^ reductases that accept electrons from quinone^10^. The generated NO_2_^−^ is further converted to NH_3_ by nitrite reductases (NIRs) using quinone as electron donors. Among various NIRs, the Cu-type NIRs (Cu-NIRs) found in Rhizobium is one of the most important enzymes for N_2_ fixation. Cu-NIRs are trimeric proteins composed of 3 identical subunits, and each monomer has two types of Cu atomic active centers, acting as electron-donating centers (T1Cu) and catalytic centers (T2Cu), respectively^10^. The reported mechanism proposes that *NO_2_^−^ (where * denotes an adsorbed specie) is bound to the T2Cu in a bidentate form via two oxygen atoms. Due to the electrons transfer from T1Cu to T2Cu, the T2Cu oxidation state decreases from (II) to (I), facilitating *NO_2_^−^ association with T2Cu in a bridging nitro binding form. Meanwhile, the aspartate acid besides the T2Cu provides proton to one oxygen and extends the N-O bond length, leading to the breaking of the N-O bond^11^. According to the mechanism of NO_2_^−^ reduction on T2Cu and T1Cu, it can be speculated that moderate affinity with NO_3_^−^, protons availability and electrons provision are the key factors for the high efficiency in NO_3_^−^RR to NH_3_. Jimmy C. Yu et al.^12^ proved that hydrogen adsorption (*H), with moderate adsorption energy on catalysts, can behave as an important reactive species for the hydrogenation of NO_x_ intermediates to NH_3_ whilst suppressing the hydrogen evolution reaction (HER) competition. Matthew J. Liu et al.^13^ also found that the NO_3_^−^RR activity and product selectivity highly depended on the *H coverage on the titanium surface at different potentials with the increase of NO_3_^−^RR overpotential.

Very recently, Schuhmann and his colleagues^14^ reported a tandem mechanism for PO_4_^3−^-modified CuCo binary metal sulfides. They proposed that NO_2_^−^ intermediates were preferentially formed on Cu-based phases followed by splitting over nearby Co-based phases; however, the role of *H during NO_3_^−^RR was unclear and the effect of the electronic structure of alloyed catalysts on the adsorption of reaction intermediates lacked further discussions in the proposed mechanism^14^. In this study, inspired by the bifunctional nature of the Cu-NIRs, we prepared CuCo alloy nanosheets via a one-step electrodeposition route. The CuCo alloy could well mimic the behavior of the two catalytic centers in Cu-NIRs. The Co species could efficiently provide the electrons and generate the hydrogen protons to the nearby Cu species with high adsorption of NO_3_^−^ and its derivatives. The as-prepared CuCo nanosheet exhibited superior catalytic performances, as (1) the lowest η of 290 mV for ammonia production (at 0.4 V *vs.* reversible hydrogen electrode (RHE), all electrode potentials mentioned below were provided with respect to RHE unless specially stated); (2) an ampere-level current density of 1035 mA cm^−2^ with a 100% Faradaic efficiency for NH_3_ generation at an overpotential of 890 mV, and a corresponding ${{{{{\rm{Yield}}}}}}_{{{{{{\rm{NH}}}}}}_{3}}$ of 4.8 mmol cm^−2^ h^−1^; (3) a wide potential window (300 mV, from −0.1 to −0.4 V) for NH_3_ generation with >90% Faradaic efficiency (FE), which is one of the most advanced catalysts for NO_3_^−^RR so far. Electrochemical in situ Fourier transform infrared spectroscopy (FTIR) and in situ shell-isolated nanoparticle enhanced Raman spectroscopy (SHINERS) associated with density functional theory (DFT) calculations were conducted to clarify the pathways and mechanisms of NO_3_^−^RR, with the aim to contribute to subsequent catalyst optimization and scaling up.

## Results

### Preparation and characterization of CuCo bimetallic electrocatalysts

**Fig. 1:**
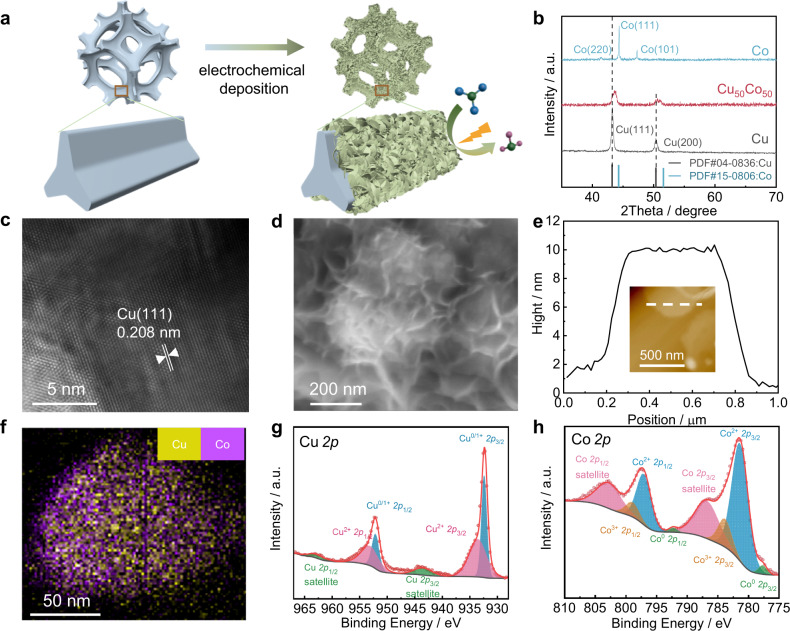
Preparation strategy and characterization of catalysts. **a** Schematic diagram of CuCo alloy electrodeposition on nickel foam’s surface. **b** XRD spectra of Cu_50_Co_50_, Cu, and Co. HRTEM image (**c**), SEM image (**d**), linear topography profiles from AFM images (**e**), and EDS mapping analysis (**f**) of Cu_50_Co_50_. XPS peaks spectra of Cu 2*p* (**g**) and Co 2*p* (**h**) of Cu_50_Co_50_.

CuCo bimetallic materials were synthesized by co-electrodeposition of Cu and Co on Ni foams’ surfaces (Fig. 1a and Method section). Ni foam is widely used as a supporting substrate for nanostructured electrocatalysts due to its smooth surface and good conductivity, benefiting the electrodeposition by an efficient electron transfer^15–17^. Meanwhile, Ni was proved as a relatively inert material for NO_3_^−^RR^18,19^, without affecting the CuCo catalysts’ performance. The Cu/Co molar ratio was determined using an inductively coupled plasma-optical emission spectrometer (ICP-OES) (Supplementary Table S1). The catalyst with a Cu/Co molar ratio of *ca*. 50/50 was named Cu_50_Co_50_ and was considered as a reference in this study. The alloying of Cu and Co in Cu_50_Co_50_ was confirmed by X-ray diffraction (XRD) and high-resolution transmission electron microscopy (HRTEM). Indeed, in the diffractogram (Fig. 1b and Supplementary Fig. S1) shifts in the Cu (111) and Cu (200) diffraction peaks toward higher degrees were observed after the addition of Co to Cu, which were attributed to the shrinkage of the lattice spacing caused by the partial alloying of Co atom with a smaller diameter compared to Cu atom^20^. Furthermore, the lattice spacing of the Cu (111) plane contracted to 0.208 nm after alloying Cu with Co (Fig. 1c), when it was 0.212 nm for the Cu (111) plane for the pure Cu catalyst (Supplementary Fig. S2a)^15^. Scanning electron microscopy (SEM) was applied to examine the morphologies of Cu, Co and Cu_x_Co_y_ catalysts and showed micro-pines structure for all the catalysts (Supplementary Fig. S3a–j). At the nanoscale level, small bump structures on the surface of the micro-pines were observed on pure Cu (Supplementary Fig. S3a, b). After the incorporation of Co, a nanosheet structure emerged on the micro-pine’s surface of the Cu_50_Co_50_ catalyst (Fig. 1d and Supplementary Fig. S3e, f), very similar to the structure of pure Co (Supplementary Fig. S3i, j). The thickness of the Cu_50_Co_50_ nanosheet was evaluated by atomic force microscopy (AFM) and was around 10 nm (Fig. 1e). Besides, the EDS mapping analysis disclosed an even distribution of Cu and Co in Cu_50_Co_50_ (Fig. 1f).

The electronic properties of the Cu_50_Co_50_ nanosheet were explored by X-ray photoelectron spectroscopy (XPS). Cu^2+^ 2*p* peaks were observed in the high-resolution Cu 2*p* spectra (Fig. 1g), and was due to the partial oxidation of the alloy’s surface exposed to air, and the same observation was made for Co (Fig. 1h). The decrease of the Cu 2*p* binding energy, compared with pure Cu (Supplementary Fig. S4a) and the notable increase of the Co 2*p* binding energy, compared with pure Co (Supplementary Fig. S4b), revealed a redistribution of the electrons between Cu and Co after their alloying^21^, leading to the movement of the *d* band towards the Fermi level^22^ comparing with the pure Cu catalyst. X-ray absorption spectroscopy (XAS) analysis was also performed to check the redistribution of electrons between Cu and Co. Extended X-ray absorption fine structure (XANES) spectra depicted a negative shift of the absorption edge position for the Cu K-edge after interacting with Co (indicated by the red arrow in the inset) (Supplementary Fig. S5), illustrating the electron density transfer from Co to Cu^23^. In addition, according to the Bader charge analysis (Supplementary Fig. S6), compared with monometallic Cu and Co, a charge redistribution on CuCo(111) was observed, where the Cu center displayed a higher electrons density compared to the Co center (Supplementary Fig. S7)^24^. The correlation between electrons redistribution within Cu and Co and the adsorption energy of *H, *NO_3_ and the *NO_x_ intermediates^25^ are discussed further in the mechanistic study section.

### Electrochemical activity and kinetics of NO_3_^−^RR

**Fig. 2:**
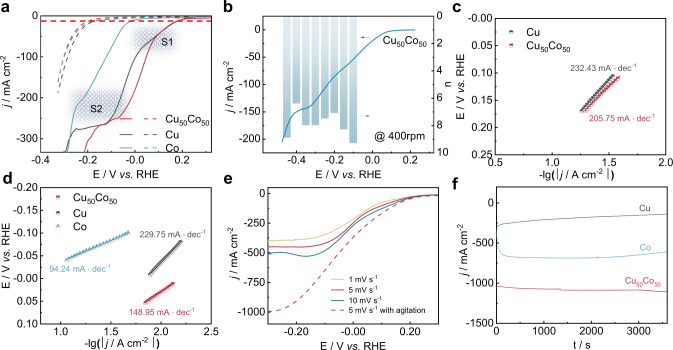
Electrochemical responses of Cu_50_Co_50_, pure Cu, and pure Co catalysts. **a** *j*-*E* curve (80% i*R* corrected) over Cu_50_Co_50_, pure Cu, and pure Co modified Ni foams (catalysts loading was 5 mg cm^−2^) in 1 M KOH solution containing 100 mM KNO_3_ (solid lines) or in the absence of KNO_3_ (dotted line) at a scan rate of 1 mV s^−1^ (the red dash line presenting the *j* of 10 mA cm^−2^, the shading S1 and S2 presenting the peak around 0.2 to 0.05 V and 0.05 to −0.15 V, respectively). **b** *j*-*E* curve (80% i*R* corrected) at 400 rpm and electron transfer numbers at different potentials calculated by the K–L equation for Cu_50_Co_50_ on RDE in 100 mM KNO_3_ + 1 M KOH electrolyte at a scan rate of 10 mV s^−1^ (catalysts loading was 0.25 mg cm^−2^). Tafel slopes in the potential range of peak S1 (**c**) S2 (**d**)**. e** *j*-*E* curves over Cu_50_Co_50_ modified Ni foam in 1 M KOH solution containing 100 mM KNO_3_ at different scan rates without agitation (solid line) and at a scan rate of 5 mV s^−1^ with agitation (catalysts loading was 5 mg cm^−2^). **f** Time-dependent current density curves over Cu_50_Co_50_, Cu, Co modified Ni foam at −0.2 V with a magnetic stirring speed of 1000 rpm (catalysts loading was 5 mg cm^−2^).

NO_3_^−^RR was first investigated by linear sweep voltammetry (LSV) at a low scan rate of 1 mV s^−1^ on all the prepared electrocatalysts. The reduction of NO_x_ species contributed mainly to the current density (solid curve), indicating a high catalytic activity toward NO_3_^−^RR for all the catalysts (Fig. 2a). The substrate of Ni foam was inactive for NO_3_^−^RR compared with the electrodeposited catalysts (Supplementary Figs. S8, S9). The overpotential η at 10 mA cm^−2^ (denoted ${\eta }_{{@{{{{{\rm{10mA}}}}}}{{{{{\rm{cm}}}}}}}^{-2}}$, η = *E*^o^ – *E*, where *E* is the potential with a current density of 10 mA cm^-2^, *E*^o^ = 0.69 V), where NO_3_^−^RR is under kinetic control, was used as a criterion to compare the catalysts’ activity. Cu_50_Co_50_ and Cu exhibited a lower energy barrier for NO_3_^−^RR, with ${\eta }_{{@{{{{{\rm{10mA}}}}}}{{{{{\rm{cm}}}}}}}^{-2}}$ of 498 and 503 mV, respectively, compared to Co, that displayed a ${\eta }_{{@{{{{{\rm{10mA}}}}}}{{{{{\rm{cm}}}}}}}^{-2}}$ of 690 mV. In fact, two reduction current peaks (peak S1 and S2) were observed in the curves of Cu and Cu_50_Co_50_. At the initial stage of the reaction, Cu and Cu_50_Co_50_ seemed to display a similar behavior toward NO_3_^−^RR, suggesting the important role of Cu at this stage. According to previous studies^26,27^, the peak S1 near 0.08 V was assigned to *NO_3_^−^ (adsorbed NO_3_^−^) reduction into *NO_2_^−^ (1) following a 2-electrons transfer process, while the peak S2 was allocated to the *NO_2_^−^ reduction into *NH_3_ (2) following a 6-electrons transfer process. In the peak S2 region, Cu_50_Co_50_ displayed a positive potential shift of 67 mV, compared to Cu, suggesting a significant synergy between Co and Cu and a drop of the barrier energy of *NO_2_^−^ reduction to *NH_3_, as the applied potential increases.

1 $${*{{{{\rm{NO}}}}}}_{3}^{-}+2{{{{{\rm{e}}}}}}^{-}+{{{{{\rm{H}}}}}}_{2}{{{{\rm{O}}}}}\to {*{{{{\rm{NO}}}}}}_{2}^{-}+2{{{{{\rm{OH}}}}}}^{-}$$

2 $$*{{{{{\rm{NO}}}}}}_{2}^{-}+6{{{{{\rm{e}}}}}}^{-}+5{{{{{\rm{H}}}}}}_{2}{{{{\rm{O}}}}}\to {*{{{{\rm{NH}}}}}}_{3}+7{{{{{\rm{OH}}}}}}^{-}$$

In the aim to investigate the reaction routes for all the catalysts, the number of electrons transferred (n) during the NO_3_^−^ reduction reaction was estimated from the slope of the Koutecký–Levich (K–L) plots (Supplementary Fig. S10). For the Cu catalyst (Supplementary Fig. S11a), n was 2 at −0.1 V and a quasi-first-order reaction relationship between *j* and the NO_3_^−^ concentration was obtained (Supplementary Fig. S12), validating the rate-determinate step (RDS) as the reduction route described by Eq. (1). The n value increased to 6 at −0.25 V, validating the reduction route described in Eq. (2). In comparison, a direct 8-electrons transfer process was observed on Cu_50_Co_50_ in the potential region between the peaks S1 and S2 (Fig. 2b), revealing a strong alloying effect promoting simultaneously both routes. Though an 8-electrons transfer process also occurred on the pure Co catalyst at a far less negative potential of *ca*. −0.45 V (Supplementary Fig. S11b), i.e., at a higher barrier energy.

In the potential region of peak S1, the Tafel slopes derived from the *j*-*E* curves (Fig. 2c), for Cu_50_Co_50_ was 205.75 mV decade^−1^, which was slightly lower than Cu (232.43 mV decades^−1^), indicating that the addition of Co could promote the electrons transfer over the catalyst/electrolyte interface during *NO_3_^−^ reduction to *NO_2_^−^. This result was supported by EIS data (Supplementary Fig. S13), demonstrating a smaller charge-transfer resistance on Cu_50_Co_50_ compared to Cu (3.51 vs. 3.81 Ω). The Co catalyst had minor electrocatalytic activity for NO_3_^−^ reduction at this relatively positive working potential, so its Tafel slop in this potential region was not determined. In the potential region of peak S2, the Tafel slope (Fig. 2d) of Cu_50_Co_50_ (148.95 mV decades^−1^) was significantly lower than Cu (229.75 mV decades^−1^); this was explained by an electron redistribution between Cu and Co over Cu_50_Co_50_ and was emphasized in the characterization section. The Co catalyst displayed the lowest Tafel slope value of 94.24 mV decades^−1^, but at a more negative potential, i.e., 95 mV shift, than Cu_50_Co_50_. This latter result suggested that after crossing a high energy barrier, Co displayed better kinetics’ performances toward the NO_3_^−^RR for NH_3_ production compared to the Cu-based catalysts. Alloying Co to Cu implemented a faster electron transfer rate and improved kinetics’ performances toward the NO_3_^−^RR. In addition to being affected by the properties of the catalysts, the NO_3_^−^RR was also diffusion-controlled (Fig. 2e). To further evaluate the NO_3_^−^RR activity of the catalysts under steady-state conditions, potentiostatic electrolytic reduction of NO_3_^−^ in a homemade H-type electrolytic cell (Supplementary Fig. S14) was carried out by electrodepositing the catalysts on Ni foams’ surfaces. A magnetic stirring rate of 1000 rpm was used to minimize the effect of diffusion and fresh electrolyte solution was constantly supplemented to maintain a constant NO_3_^−^ ion concentration. In these conditions, the current densities obtained on the Cu_50_Co_50_ catalyst reached an Ampere level at −0.2 V *vs.* RHE (Fig. 2f).

**Fig. 3:**
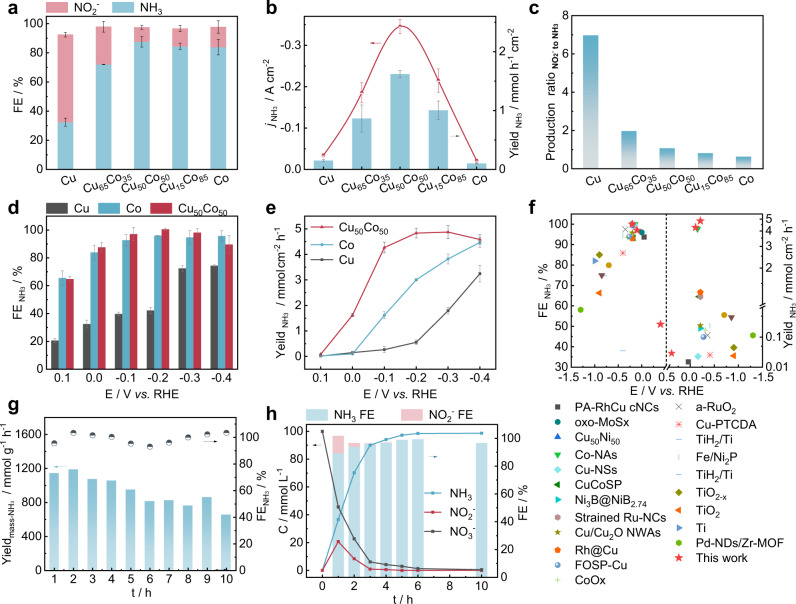
Electrochemical performance of catalysts. ${{{\mbox{FE}}}}_{{{{\mbox{NH}}}}_{3}}$and ${{{\mbox{FE}}}}_{{{{\mbox{NO}}}}_{2}^{-}}$ of NO_3_^−^RR (**a**), bias-current density and products yield for NH_3_ (**b**), and the ratio of NO_2_^−^ to NH_3_ generated (**c**) for different Cu/Co ratio at 0 V in 100 mM KNO_3_ + 1 M KOH electrolyte (catalysts loading was 5 mg cm^−2^). ${{{\mbox{FE}}}}_{{{{\mbox{NH}}}}_{3}}$ (**d**) and NH_3_ product yield (**e**) at different electrode potentials on Cu_50_Co_50_, pure Cu and pure Co catalysts modified Ni foam (catalysts loading was 5 mg cm^−2^). Comparison of the electrocatalytic NO_3_^-^RR performances of Cu_50_Co_50_ modified Ni foam with other extensively reported electrocatalysts (**f**). ${{{\mbox{FE}}}}_{{{{\mbox{NH}}}}_{3}}$ and ${{{\mbox{Yield}}}}_{{{\mbox{mass}}}-{{{\mbox{NH}}}}_{3}}$ on Cu_50_Co_50_/Ni foam under the applied potential of −0.2 V during 10 periods of 1 h electrocatalytic NO_3_^-^RR (**g**) (catalysts loading was 5 mg cm^−2^). The time-dependent concentration of NO_3_^−^, NO_2_^−^ and NH_3_ and corresponding FE over Cu_50_Co_50_ modified Ni foam at −0.1 V (**h**) (catalysts loading was 5 mg cm^−2^). Error bars represent the standard deviations calculated from three independent measurements.

NH_3_ and NO_2_^−^ were quantitatively detected by the Nessler Reagent method and ion chromatography, respectively (Supplementary Fig. S15)^28,29^. The gas products were analyzed during the reaction by gas chromatography and online electrochemical mass spectrometry (OEMS)^30^. There was no N_2_ production detected and the amount of the stripped NH_3_ in this work was negligible. (Supplementary Fig. S16) In order to figure out the effect of Co content on the NO_3_^−^RR activity, CuCo bimetallic catalysts with different Cu/Co ratios were prepared, i.e., Cu_65_Co_35_ and Cu_15_Co_85_. As shown in Fig. 3a, when a potential of 0 V was applied, the Faraday efficiency of NH_3_ (${{{{{\rm{FE}}}}}}_{{{{{{\rm{NH}}}}}}_{3}}$) and NO_2_^−^ (${{{{{\rm{FE}}}}}}_{{{{{{\rm{NO}}}}}}_{2}^{-}}$) on pure Cu were 32% and 60%, respectively. The corresponding molar ratio of NO_2_^−^ to NH_3_ was seven times higher (Fig. 3c), suggesting an accumulation of NO_2_^−^ due to the low kinetic of *NO_2_^−^ hydrogenation to *NH_3_ on the Cu surface. With the addition of Co to Cu, both the ${{{{{\rm{FE}}}}}}_{{{{{{\rm{NH}}}}}}_{3}}$ and the geometrically normalized current density of NH_3_ production (${j}_{{{{{{\rm{NH}}}}}}_{3}}$) increased significantly (Fig. 3a, b). A volcano shape was observed and the highest ${j}_{{{NH}}_{3}}$ was obtained on Cu_50_Co_50_ (i.e., 347 mA cm^−2^ ${j}_{{{{{{\rm{NH}}}}}}_{3}}$ and 88% ${{{{{\rm{FE}}}}}}_{{{{{{\rm{NH}}}}}}_{3}}$), and was ten times higher than monometallic Cu (i.e., 34 mA cm^−2^ and 32% ${{{{{\rm{FE}}}}}}_{{{{{{\rm{NH}}}}}}_{3}}$), and nearly 17 times higher than monometallic Co catalyst (i.e., 21 mA cm^−2^ and 84% ${{{{{\rm{FE}}}}}}_{{{{{{\rm{NH}}}}}}_{3}}$). Furthermore, the molar ratio of NO_2_^−^ to NH_3_ exhibited a dramatic drop from 7 to 0.6 as the Co content was raised from 0 to 100% (Fig. 3c). This observation suggested that the incorporation of Co enhanced further the *NO_2_^−^ hydrogenation into NH_3_. Moreover, when the ratio of Co was increased by over 50%, the ${{{{{\rm{FE}}}}}}_{{{{{{\rm{NH}}}}}}_{3}}$ was remained constant, but the ${{{{{\rm{j}}}}}}_{{{{{{\rm{NH}}}}}}_{3}}$ declined dramatically, indicating that a moderate Cu/Co ratio was important to maintain high catalytic performance for NH_3_ production. The electrochemically active surface area (ECSA) was also measured (Supplementary Figs. S17f, S18b), and all the catalysts had comparable ECSA. The maximum ECSA normalized current density for NH_3_ production (${j}_{{{{{{\rm{NH}}}}}}_{3}({{{{\rm{ECSA}}}}})}$) was obtained on Cu_50_Co_50_ (Supplementary Fig. S19), indicating that this catalyst had the highest intrinsic activity for NH_3_ production.

To clarify the possible ^14^N pollution, the electrolysis in the electrolyte free of NO_3_^−^ ions was performed and little NH_3_ was produced (Supplementary Fig. S20). ${j}_{{{{{{\rm{NH}}}}}}_{3}}$ in the solution with NO_3_^−^ ions was over 150-fold higher than that in the electrolyte free of NO_3_^−^. The content of ^14^NH_4_^+^ was also analyzed by ^1^H NMR test^29,31^ and was *ca*. 4.62 mmol cm^−2^ h^−1^. The obtained results were close to the one from the spectrophotometric analysis (4.75 mmol cm^−2^ h^−1^) (Supplementary Fig. S21d), and confirmed the reliability of the quantification methods used in this work. The typical two peaks of ^15^NH_4_^+^ after the electrolysis of ^15^NO_3_^−^ also suggested that the NH_3_ product indeed came from the electrocatalytic reduction of NO_3_^−^ (Supplementary Fig. S21e).

It is known that the applied potential influences the products’ selectivity^13^, so we investigated the effect of the applied potential toward the NO_3_^−^RR (Fig. 3d, e and Supplementary Fig. S22). The ${{{{{\rm{FE}}}}}}_{{{{{{\rm{NH}}}}}}_{3}}$ was about 51% at a η of 290 mV (at 0.4 V) on Cu_50_Co_50_. The η was comparatively much lower than that of the state-of-the-art catalysts reported in the literature (Fig. 3f and Supplementary Table S2). When the η reached 590 mV (at 0.1 V), the ${{{{{\rm{FE}}}}}}_{{{{{{\rm{NH}}}}}}_{3}}$ got about 65% on Cu_50_Co_50_ (Fig. 3d and Supplementary Fig. S23). In comparison, the ${{{{{\rm{FE}}}}}}_{{{{{{\rm{NH}}}}}}_{3}}$ was around 21% on Cu at 0.1 V (Fig. 3d), with NO_2_^−^ as the main product. As the electrode potential shifted negatively, the intermediate NO_2_^−^ was rapidly reduced (Supplementary Fig. S23). At −0.2 V, the ${{{{{\rm{FE}}}}}}_{{{{{{\rm{NH}}}}}}_{3}}$ on Cu_50_Co_50_ achieved 100 ± 1%, and ${j}_{{{{{{\rm{NH}}}}}}_{3}}$ reached 1035 mA cm^−2^ (Supplementary Fig. S24b), corresponding to an NH_3_ production rate of 4.8 mmol cm^−2^ h^−1^ that is about two and eight times higher than the ones obtained on monometallic Co and Cu (Fig. 3e and Supplementary Fig. S25), respectively. Based on the charge consumed during the CuCo electrodeposition on the Ni foam substrate, the mass activity of NH_3_ yield (${{{{{\rm{Yield}}}}}}_{{{{{\rm{mass}}}}}-{{{{{\rm{NH}}}}}}_{3}}$) on Cu_50_Co_50_ was estimated roughly to 960 mmol g_cat_^−1^ h^−1^ at 0.2 V. The obtained ${{{{{\rm{Yield}}}}}}_{{{{{\rm{mass}}}}}-{{{{{\rm{NH}}}}}}_{3}}$ was slightly underestimated since hydrogen evolution was observed during CuCo electrodeposition. It should be noted that the production of NH_3_ from electrocatalytic NO_3_^−^RR is currently at a laboratory scale. More follow-up pilot tests and scale-up work are required to meet the industrial demands. Based on the preliminary calculations, we consider that our work inspired by the bifunctional nature of nitrite reductase, provides a new expectation and shows a great prospect, and after further development could compete with the well-established Haber–Bosch process^12,32^ which currently shows a ${{{{{\rm{Yield}}}}}}_{{{{{\rm{mass}}}}}-{{{{{\rm{NH}}}}}}_{3}}$ of ca. 200 mmol g_cat_^−1^ h^−1^ at industrial scale. The potential window for ${{{{{\rm{FE}}}}}}_{{{{{{\rm{NH}}}}}}_{3}}$ above 90% was wide and ranged from −0.1 to −0.4 V (Fig. 3d). Ten cycles of electrolysis at constant potential were performed to check the stability of Cu_50_Co_50_ (Fig. 3g) and the results displayed a stable ${{{{{\rm{FE}}}}}}_{{{{{{\rm{NH}}}}}}_{3}}$ exceeding 90% over the cycles. According to the SEM analysis, the decay of the yield for NH_3_ would be due to the nanosheet agglomeration after the consecutive recycling tests (Supplementary Fig. S26a, b). Furthermore, XRD (Supplementary Fig. S26c) and XPS (Supplementary Fig. S26d, e) analysis was also performed on the samples after the consecutive recycling tests, and the results demonstrated negligible changes in the chemical compositions and oxidation states, which confirmed the excellent stability of the catalyst.

The concentration of NO_3_^−^ in real wastewater can vary from 0.88 mmol L^−1^ to 1.95 mol L^−1^ ^33^. Therefore, NO_3_^−^RR on Cu_50_Co_50_ was performed at a wide range of NO_3_^−^ concentrations (1–100 mmol L^−1^). The ${{{{{\rm{FE}}}}}}_{{{{{{\rm{NH}}}}}}_{3}}$ was maintained above 95% in the whole range of NO_3_^−^ concentrations (Supplementary Fig. S27) at −0.1 V. The NO_3_^−^RR in neutral conditions was carried out on Cu_50_Co_50_ catalysts at −0.2 V in an electrolyte solution of 0.1 M KNO_3_ + 0.5 M K_2_SO_4_. The current density of NO_3_^−^RR on Cu_50_Co_50_ in a neutral condition was higher than the one on monometallic Cu and Co, and the ${{{{{\rm{FE}}}}}}_{{{{{{\rm{NH}}}}}}_{3}}$ over Cu_50_Co_50_ was more than 90% (Supplementary Fig. S28). These experiments demonstrated the excellent property of Cu_50_Co_50_ toward NO_3_^−^ recovery in various environmental wastewater systems. In batch conditions with an initial nitrate’s concentration of 100 mmol L^−1^ (*ca*. 6200 ppm) at a reduction potential of −0.1 V and after 10 h, the nitrate’s removal efficiency reached 99.5%, with a ${{{{{\rm{FE}}}}}}_{{{{{{\rm{NH}}}}}}_{3}}$ of 96% (Fig. 3h). The remaining NO_3_^−^ in the solution was 31 ppm which was much lower than the limitations fixed by the World Health Organization for drinking water, (i.e., 50 ppm)^34^. Several processes can be then considered for further extracting NH_3_, such as air stripping, ion exchange, struvite precipitation, etc.^35^.

Electrochemical in situ FTIR, SHINERS and DFT calculations were conducted to elucidate the reaction mechanism as well as the origin of the different activities observed between the catalysts.

### Electrochemical in situ FTIR analysis of NO_3_^−^RR

**Fig. 4:**
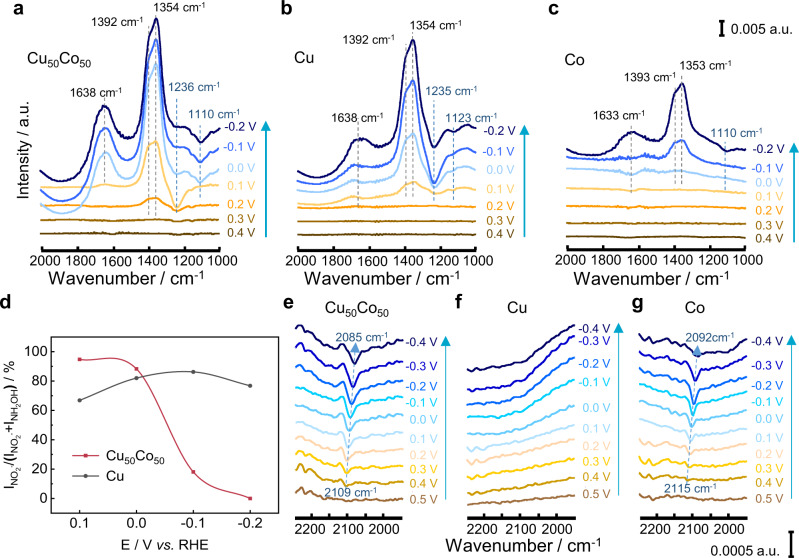
Electrochemical in situ FTIR spectra. Electrochemical thin-layer in situ FTIR spectra of NO_3_^−^RR on Cu_50_Co_50_ (**a**), Cu (**b**), and Co (**c**) in 100 mM KNO_3_ + 1 M KOH. **d** $\frac{{{{{{\rm{I}}}}}}_{{{{{{\rm{NO}}}}}}_{2}^{-}}}{{{{{{\rm{I}}}}}}_{{{{{{\rm{NH}}}}}}_{2}{{{{\rm{OH}}}}}}+{{{{{\rm{I}}}}}}_{{{{{{\rm{NO}}}}}}_{2}^{-}}}$ ratio at different electrode pot**e**ntials. ATR-FTIR spectra on Cu_50_Co_50_ (**e**), Cu (**f**), and Co (**g**) in 1 M KOH.

The electrochemical thin-layer in situ FTIR can track intermediates in solution within the thin-layer (thickness around 10 μm) between the electrode and IR window and species adsorbed on the electrode surface^36^. A reference spectrum (R_Ref_) at reference potential (*E*_R_, 0.4 V) was firstly acquired, and then the potential was stepped to studied potentials (*E*_S_) and to collect working spectra (R_S_). The resulting spectra were represented as relative changes in the reflectance: ΔR/R = (R_S_-R_ref_)/R_Ref_. As a result, the downward band in the resulting spectra indicated the formation of NO_3_^−^ intermediates at *E*_S_, while the upward band referred to the consumption of NO_3_^−^. The FTIR peaks observed on Cu_50_Co_50_, Cu, and Co are compiled in Supplementary Table S3. As illustrated in Fig. 4, in the potential range from 0.4 to −0.2 V, the absorption bands were assigned to intermediates present in the electrolyte, since the wavenumbers of all the absorption bands were independent of the working potential^37^. In Fig. 4a, five obvious absorption bands appeared in the infrared spectra of Cu_50_Co_50_ viz. (1) At the working potential of 0.2 V, close to the onset potential of the LSV curve, the upward absorption bands at 1392 and 1354 cm^−1^ were ascribed respectively to N-O symmetric and asymmetric stretching vibration of NO_3_^−^^38^, indicating consumption of NO_3_^−^ species in the thin layer; (2) at the same time, the downward band at 1236 cm^−1^ appeared and was attributed to N-O antisymmetric stretching vibration of NO_2_^−^^39^, indicating NO_2_^−^ formation from NO_3_^−^ reduction; (3) with potential negatively moving to 0.1 V, another intermediate observed around 1110 cm^−1^ was ascribed to -N-O- stretching vibration of hydroxylamine (NH_2_OH)^39,40^, which was a key intermediate for NH_3_ formation; (4) The upward band around 1638 cm^−1^ was attributed to water electrolysis responsible of hydrogen generation involved in the hydrodeoxidation of NO_3_^−^ in the solution of thin-layer^41^.

The FTIR spectra collected on Cu (Fig. 4b) were very similar to the Cu_50_Co_50_ catalyst’s ones, indicating that the NO_3_^−^RR behaviors were similar for both catalysts. However, the NO_3_^−^ consumption on Cu at a potential of 0.1 V was 100 mV more negative than the one obtained on Cu_50_Co_50_, indicating a better kinetic with the latter one. In addition, with the negative shift of the working potential, the band intensity of NO_2_^−^ relative to the sum intensity of NO_2_^−^ and NH_2_OH production (i.e.,$\frac{{{{{{\rm{I}}}}}}_{{{{{{\rm{NO}}}}}}_{2}^{-}}}{{{{{{\rm{I}}}}}}_{{{{{{\rm{NH}}}}}}_{2}{{{{\rm{OH}}}}}}+{{{{{\rm{I}}}}}}_{{{{{{\rm{NO}}}}}}_{2}^{-}}}$) on Cu_50_Co_50_ dropped down sharply, while the $\frac{{{{{{\rm{I}}}}}}_{{{{{{\rm{NO}}}}}}_{2}^{-}}}{{{{{{\rm{I}}}}}}_{{{{{{\rm{NH}}}}}}_{2}{{{{\rm{OH}}}}}}+{{{{{\rm{I}}}}}}_{{{{{{\rm{NO}}}}}}_{2}^{-}}}$ ratio was almost independent of the potential on Cu (Fig. 4d). The two phenomena, i.e., the appearance of NH_2_OH after the formation of NO_2_^−^ and the increase of NH_2_OH at the expense of the consumption of NO_2_^−^, suggested that *NH_2_OH was obtained by the deep hydrogenation of *NO_2_^42^. The $\frac{{{{{{\rm{I}}}}}}_{{{{{{\rm{NO}}}}}}_{2}^{-}}}{{{{{{\rm{I}}}}}}_{{{{{{\rm{NH}}}}}}_{2}{{{{\rm{OH}}}}}}+{{{{{\rm{I}}}}}}_{{{{{{\rm{NO}}}}}}_{2}^{-}}}$ ratio on Cu_50_Co_50_ was much lower than the one on Cu catalyst, demonstrating that alloying Cu with Co could deeply enhance the hydrogenation of *NO_2_ to the final product NH_3_ on Cu/Co alloy catalysts. Thereby we speculated that Co sites were responsible for the variation of the $\frac{{{{{{\rm{I}}}}}}_{{{{{{\rm{NO}}}}}}_{2}^{-}}}{{{{{{\rm{I}}}}}}_{{{{{{\rm{NH}}}}}}_{2}{{{{\rm{OH}}}}}}+{{{{{\rm{I}}}}}}_{{{{{{\rm{NO}}}}}}_{2}^{-}}}$ ratio, owing to their excellent protons’ adsorption (*H) capacity, as it will be demonstrated in the next section, promoting the hydrodeoxidation of *NO_2_ to *NH_2_OH according to Eqs. (3) and (4):^43^

3 $${*{{{{{\rm{NO}}}}}}}_{2}+2*{{{{{\rm{H}}}}}}\to*{{{{{\rm{NO}}}}}}+{{{{{{\rm{H}}}}}}}_{2}{{{{{\rm{O}}}}}}$$

4 $$*{{{{{\rm{NO}}}}}}+3*{{{{{\rm{H}}}}}}\to*{{{{{{\rm{NH}}}}}}}_{2}{{{{{\rm{OH}}}}}}$$

On pure Co (Fig. 4c), no spectra bands of NO_3_^−^RR were detected until the working potential negatively shifted to 0 V. The absence of NO_2_^−^ on the Co catalyst suggested a low NO_2_^−^ accumulation in the thin layer solution, corroborating very well first the results obtained from the K–L equation where the Co catalyst was more inclined to perform continuous hydrogenation of NO_3_^−^ to NH_3_ via an 8-electrons transfer and second the lower Tafel slope in the Peak S2 region data.

In comparison with thin-layer in situ FTIR, attenuated total reflection in situ FTIR analysis (ATR-FTIR) is more sensitive to the signal of adsorbed species on catalysts’ surfaces^44^. Two weak vibration bands of adsorbed NO in two different adsorption modes (“bridge” and “on top”) were detected at 1557 and 1639 cm^−1^ on the Cu_50_Co_50_ catalyst (Supplementary Fig. S29a)^38,45^. Interestingly, a downward band center at 2109 cm^−1^ at 0.5 V was observed in NO_3_^–^free electrolyte on Cu_50_Co_50_ (Fig. 4e), and the band center was negatively shifted to 2085 cm^−1^ at −0.4 V, yielding a Stark turn rate of 26.7 cm^−1^ V^−1^. These IR features could be attributed to adsorbed *H on the Cu_50_Co_50_ surface, and indicated also the enhancement of *H adsorbed on the Cu_50_Co_50_ surface at a higher η, which is consistent with the reported studies^46,47^. The fact that *H was observed on Cu_50_Co_50_ and Co (around 2110 cm^−1^) (Fig. 4e, g and Supplementary Fig. S30c) while being absent on Cu (Fig. 4f and Supplementary Fig. S30b), indicated that *H on Cu_50_Co_50_ was mainly attributed to water dissociation on Co sites. When the potential negatively shifted, the intensity of Co-H gradually raised, demonstrating that more *H were generated which in turn enhanced the hydrodeoxidation reaction and gave a lower $\frac{{{{{{\rm{I}}}}}}_{{{{{{\rm{NO}}}}}}_{2}^{-}}}{{{{{{\rm{I}}}}}}_{{{{{{\rm{NH}}}}}}_{2}{{{{\rm{OH}}}}}}+{{{{{\rm{I}}}}}}_{{{{{{\rm{NO}}}}}}_{2}^{-}}}$ ratio value. The wavenumber attributed to *H on Co was slightly higher than the one on Cu_50_Co_50_, indicating a stronger affinity of *H for pure Co catalyst. In the presence of NO_3_^−^, Co-H was still present in the spectra of pure Co (Supplementary Fig. S30c), while it was vanished in the spectra of Cu_50_Co_50_ (Supplementary Fig. S30a). This could be explained by the fact that *H could adsorb quickly on Co sites inhibiting the adsorption of NO_x,_ then the adsorbed hydrogen can react with the equivalent amount of *NO_x_ on nearby Cu sites giving a high reaction kinetics. In the case of monometallic Co, the active sites are majorly occupied by *H species preventing the adsorption of NO_x_ onto the catalyst’s surface, leading to weak activity. The presence of active hydrogen (H*) in the reaction process was also verified by electron paramagnetic resonance (EPR) analysis on Cu_50_Co_50_ catalyst (Supplementary Fig. S31) using 5,5-dimethyl-1-pyrroline *N*-oxide (DMPO) as a spin trap^48^. The intensity of the EPR signal of the DMPO-H adduct on the Cu_50_Co_50_ catalyst decreased when nitrate was added into the electrolyte, indicating the consumption of active hydrogen during NO_3_^−^RR. These results were consistent with the ATR-FTIR analysis. Based on the coupling of thin-layer in situ FTIR and ATR-FTIR analysis, therefore, we proposed the following pathway for the NO_3_^−^RR on Cu_50_Co_50_: NO_3_^−^ → *NO_3_ → *NO_2_ → *NH_2_OH → *NH_3_, where *H on Co sites can promote the deep hydrodeoxidation of NO_2_^−^ to NH_2_OH.

### SHINERS analysis of NO_3_^−^RR

**Fig. 5:**
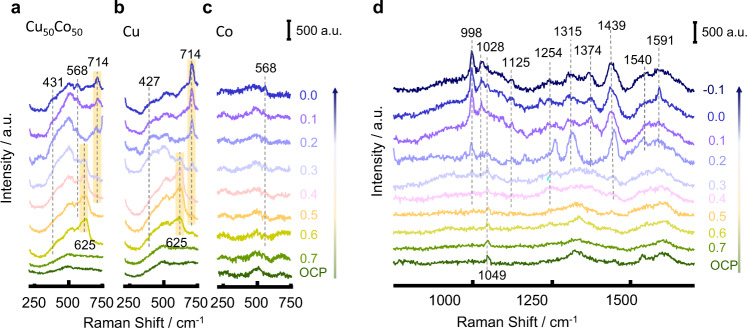
Electrochemical SHINERS spectra of NO_3_^−^RR. SHINERS spectra between 230–750 cm^−1^ on Cu_50_Co_50_ (**a**), Cu (**b**), and Co (**c**). SHINERS spectra between 750–1700 cm^−1^ on Cu_50_Co_50_ (**d**) in 100 mM KNO_3_ + 10 mM KOH during cathodic polarization from 0.7 to −0.1 V.

The reaction intermediates provided by electrochemical in situ FTIR spectra were still insufficient to figure out the overall roadmap of NO_3_^−^RR to NH_3_, in this aim, SHINERS spectra of the catalysts were collected to probe the catalysts’ surface during the reaction. Supplementary Table S4 compiled the Raman scattering peaks observed during NO_3_^−^ reduction on Cu_50_Co_50_, Cu, and Co, which were not detected on Au@SiO_2_ (Supplementary Fig. S32). The wavenumber below 750 cm^−1^ corresponds mainly to the chemical properties of the catalyst’s surface^43^. SHINERS spectra of Cu_50_Co_50_, Cu, and Co, in this section, were summarized and shown in Fig. 5. At 0.6 V, a characteristic band at 625 cm^-1^ associated with Cu_2_O^49^ was observed on Cu and Cu_50_Co_50_ (Fig. 5a, b), indicating partial oxidation of the catalysts surface due to air exposition before NO_3_^−^RR; these results were consistent with the XPS data. As the working potential decreased to 0.3 V, the band intensity of Cu_2_O gradually shrank, and a peak at 714 cm^−1^ emerged in replacement and was associated with the bending mode of free Cu-OH_ad_^50,51^, indicating a gradual reduction of Cu before NO_3_^−^RR started to occur at 0.2 V. Besides, the band at 431 cm^−1^ can be assigned to Cu-O_x_ due to the adsorption of oxynitride on Cu surface^51^, and the band’s intensity increased as the potential moved negatively. For Co (Fig. 5c), a band at 568 cm^−1^ was also observed, associated with the formation of Co-O_x_ caused by the same oxynitride species adsorbed on the catalyst’s surface^52^.

In addition, the signals of NO_3_^−^ and its reduced intermediates adsorbed on the catalysts’ surface were observed in the spectra between 750 and 1700 cm^−1^. As the potential decreased from 0.7 to −0.1 V, several peaks appeared in sequences on Cu_50_Co_50_ (Fig. 5d) viz. (1) At open circuit potential (OCP) and 0.7 V, the only obvious peak viewed at around 1049 cm^−1^ was attributed to the symmetric NO_3_^−^ stretching from solution NO_3_^−^ species^53^. (2) When the potential was decreased to 0.4 V, the NO_3_^−^ species in the solution started to adsorb on the surface of Cu_50_Co_50_ since four peaks appeared in three different forms as NO stretching vibration from the unidentate nitrate near 998 cm^−1^, symmetric and antisymmetric stretching vibration of the NO_2_ group from the chelated nitro configuration around 1125 and 1254 cm^−1^^54^, and the N=O stretching vibration of the bridged nitro group closed to 1439 cm^−1^, respectively^55^. (3) As the working potential shifted negatively further to 0.2 V, the symmetric NO_3_^−^ stretch of *NO_3_^−^ appeared around 1028 cm^−1^ ^56,57^ and symmetric bending vibrations of the HNH near 1315 and 1374 cm^−1^ were apparent;^55,57^ N=O stretch of HNO^56^ was visible around 1540 cm^−1^. (4) When the potential negatively shifted further to 0.1 V, the HNO peak near 1540 cm^−1^ disappeared quickly; meanwhile, the antisymmetric bending vibration of the HNH of NH_3_ at 1591 cm^−1^ came out^58^, which indicated an efficient formation of NH_3_ from HNO hydrodeoxidation.

The SHINERS spectra on Cu and Co catalysts (Supplementary Fig. S33a,b) were similar to the one of Cu_50_Co_50_, but a new peak close to 800 cm^−1^ related to the bending vibration of NO_2_^−^ group was only observed on Cu (Supplementary Fig. S33a)^26^. This could be due to the accumulation of NO_2_^−^ species near by the Cu surface due to its poor ability for deep NO_2_^−^ hydrodeoxidation properties. The intensity of ${\nu }_{{{{{{\rm{s}}}}}}({{{{{\rm{N}}}}}}{O}_{3}^{-})}$ from NO_3_^−^ species in solution on Co at low overpotential was similar to the one on Cu_50_Co_50_ and Cu, however, the band’s intensities of all the intermediates formed on Co were very weak (Supplementary Fig. S33b), meaning that a low amount of *NO_3_^−^ species and its derivates were adsorbed on the Co surface. It can be rationally speculated that the affinity of *H species to the Co surface was such strong that the active sites on the Co catalyst were majorly occupied by *H species leading to low coverage of *NO_3_^−^ species, bringing a ${{{{{\rm{FE}}}}}}_{{{{{{\rm{NH}}}}}}_{3}}$ close to 85% (Fig. 3b) at the expense of a low current density of NO_3_^−^RR of 25.4 mA cm^−2^ (Supplementary Fig. S24a) at 0 V. Similarly, *H species affinity for Cu surface was so weak that most active sites were occupied by *NO_3_^−^ species, leading to a relatively high current density up to 104.8 mA cm^−2^ (Supplementary Fig. S24a) and yet an unsatisfactory ${{{{{\rm{FE}}}}}}_{{{{{{\rm{NH}}}}}}_{3}}$ of 32% (Fig. 3b). Therefore only if a balanced coverage of *H and *NO_3_ species is achieved, a satisfactory ${{{{{\rm{FE}}}}}}_{{{{{{\rm{NH}}}}}}_{3}}$ and *j* can be simultaneously obtained, which is the case with Cu_50_Co_50_ where ${{{{{\rm{FE}}}}}}_{{{{{{\rm{NH}}}}}}_{3}}$ and *j* were 88% 394.6 mV respectively.

With combined electrochemical in situ FTIR and SHINERS spectroscopic analysis, the NO_3_^−^RR pathway on Cu, Co and CuCo was proposed as a series of deoxygenation reactions according to the following path, NO_3_^−^ → *NO_3_ → *NO_2_ → *NO, accompanied by a series of hydrogenation reactions: *NO → *NOH → *NH_2_OH → * NH_3_ → NH_3_.

### DFT calculations

**Fig. 6:**
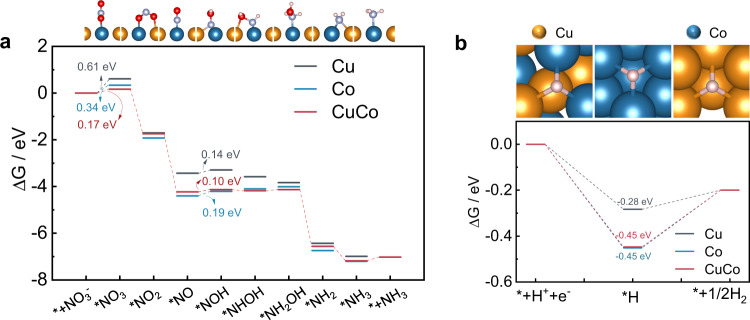
DFT calculations of NO_3_^−^RR and HER on Cu(111), Co(111), and CuCo(111). Reaction-free energies for different intermediates of NO_3_^-^RR (**a**) and HER (**b**) at −0.2 V *vs.* RHE on CuCo(111), pure Cu(111), and pure Co(111) surface, respectively.

Based on all the aforementioned observations and to shed light on the NO_3_^−^RR mechanism on Cu, Co, and CuCo at the atomic level, the density functional theory (DFT) calculations were conducted and the Gibbs free energies (ΔG) of *NO_3_ species and their derivates on Cu(111), Co(111) and CuCo(111) surface were presented in Fig. 6a.

In terms of thermodynamics, the rate-determining step (RDS) on Cu(111), Co(111) and CuCo(111) was the initial *NO_3_ adsorption step and lower energy was required on CuCo(111) (0.17 eV), compared to Cu(111) (0.61 eV) and Co(111) (0.34 eV), indicating stronger adsorption of NO_3_^−^ species on CuCo(111)^15,59^. Interestingly, the ΔG of the initial *NO_3_ species on Co(111) was lower than the one obtained on Cu(111), which should lead to better NO_3_^−^RR performances. However, this result contradicted the fact that ${\eta }_{{@10{{{{{\rm{mA}}}}}}{{{{{\rm{cm}}}}}}}^{-2}}$ of NO_3_^−^RR on Cu was 503 mV more positive than on Co (${\eta }_{{@10{{{{{\rm{mA}}}}}}{{{{{\rm{cm}}}}}}}^{-2}}$ of 690 mV). This inconformity could be explained by a strong competition of *H species on the catalyst sites’ surface compared to nitrogenous species, as proved by in situ FTIR and SHINERS analysis. Indeed, Co was capable of promoting hydrogenation during the NO_3_^−^RR by transferring in situ *H species^13^, because of its excellent adsorption for *H species^60^. However, the excessive adsorption of *H can reduce the coverage of *NO_3_ species and their intermediates, thereby weakening the electrochemical activity toward NO_3_^−^RR. Compared with the ΔG of *NO_3_ and *H on Co(111) (Fig. 6b and Supplementary Table S11), the adsorption for *H was stronger, but the adsorption for NO_3_^−^ was poor. With the incorporation of the Cu atom, the electronic structure changed, enhancing the adsorption of NO_3_^−^ species on CuCo(111) and furthermore, the partial replacement of Co sites with Cu sites also reduced the coverage of *H species.

Based on the comprehension of the results discussed above, the redistribution of electrons in Cu_50_Co_50_ facilitated the electrons transfer rate and uplifted the catalytic kinetic of the NO_3_^−^RR (Supplementary Figs. S6, S7). The fair coverage of *H and *NO_3_ species were important to obtain simultaneously a high ${{{{{\rm{FE}}}}}}_{{{{{{\rm{NH}}}}}}_{3}}$ and ${j}_{{{{{{\rm{NH}}}}}}_{3}}$. Co-adjustment of Cu and Co sites on Cu_50_Co_50_ can balance the adsorption energy between *H and *NO_3_ species, not only by lowering the energy barrier of NO_3_^−^ reduction but also by improving the hydrogenation capability with the enhanced *H adsorption compared with pure Cu, and at the same time avoiding the excessive *H occupation of the active sites compared to pure Co.

To conclude, we presented a novel Cu-based catalyst able to mimic the Cu core center of the Cu-NIR for the electrocatalytic NO_3_^−^ reduction. The addition of Co to Cu formed a micro-pine structure with nanosheets and enhanced dramatically the proton availability over the surface of the catalyst from water electrolysis, resulting in an almost full Faraday efficiency of NH_3_ production at an ampere-level current density of 1035 mA cm^−2^ at −0.2 V. This process exhibited a high activity and could be proposed as a sustainable and eco-friendly complementary route of NH_3_ production. In batch conditions, the catalyst was able to achieve a ${{{{{\rm{FE}}}}}}_{{{{{{\rm{NH}}}}}}_{3}}$ of 96% and a nitrate’s removal of 99.5%, from an initial concentration of 6,200 ppm, reaching a final concentration of 31 ppm after 10 h of reaction, lower than the limitations fixed by the World Health Organization for drinking water. A mechanism was established by combining in situ FTIR and SHINERS spectroscopic investigations and DFT calculations. The synergy between Cu and Co can reduce the high energy barrier of the rate-determining step of the initial *NO_3_ adsorption step on Cu, due to a regulation of the electronic structure. The ΔG of the hydrogenation of the intermediate species, *NO_x_, could also be reduced due to the facile adsorption of *H species on Co(111) compared to Cu(111). We proposed that a rational control of the *H adsorption over the surface of the bimetallic catalyst is the key to managing further adsorption of intermediates from NO_3_^−^RR to achieve excellent performances. This discovery can provide an additional dimension to research into surface adsorption-modulated bimetallic catalysts for highly reactive hydrodeoxidation reactions and can provide a novel strategy for the development of multi-component heterogeneous catalysts for efficient NH_3_ production and wastewater treatment.

## Methods

### Preparation of Cu_x_Co_y_, Cu, and Co catalysts

The catalysts were prepared via an electrodeposition process under the current density of 50 mA cm^−2^ for 300 s in a two-electrodes system where a platinum plate was used as the counter electrode and the Ni foam as the working electrode. Ni foams were pretreated with acetone and ethanol, and then repeatedly rinsed with ultrapure water and dried under a heating lamp. The deposition electrolyte (50 mL) comprised of 0.015 M trisodium citrate pentahydrate solutions and 50 mM CuSO_4_ + CoSO_4_^61,62^. The Cu_x_Co_y_ catalysts with Cu:Co ratios of 65:35, 50:50, and 15:85, denoted as Cu_65_Co_35_, Cu_50_Co_50_, and Cu_15_Co_85_, were obtained in the deposition solutions with the ratio of CuSO_4_ to CoSO_4_ as 60:40, 45:55, and 10:90, respectively. The Cu and Co catalysts were prepared. The preparation for pure Cu and Co catalysts followed the same steps as the Cu_x_Co_y_ synthesis, except that only CuSO_4_ or CoSO_4_ solution was present in the electroplating solution. The catalysts were finally rinsed with ultrapure water and dried under the protection of Ar.

### Material characterization

Morphology and elemental composition were characterized using a scanning electron microscope (SEM, ZEISS Sigma) with an energy dispersive X-ray spectrometer at an operating voltage of 15 kV. The lattice arrangement was observed using a high-resolution transmission electron microscope (HRTEM, FEI-Tecnai G2 F20) at an accelerated voltage of 200 kV. The chemical composition was analyzed by an inductively coupled plasma emission spectrometer (ICP-OES, Thermo Fisher iCap 7000). X-ray diffractometer (XRD, SmartLab-SE) with Cu-Kα X-ray source was used for crystal material structure analysis. Al Ka X-ray excited Thermo Fisher Scientific Nexsa X-ray Photoelectron spectrometer (XPS, Nexsa) was used for chemical state analysis. All XPS spectra were corrected with a C 1 s spectral line of 284.8 eV. X-ray absorption fine structure (XAFS) spectra at Cu K-edge and Co K-edge were obtained on the 1W1B beamline of Beijing Synchrotron Radiation Facility (BSRF) operated at 2.5 GeV and 250 mA. Standard data processing, including energy calibration and spectral normalization of the raw spectra was performed using Athena software.

### Electrochemical test

The electrochemical measurements were performed using a three-electrodes system connected to the CHI 760E workstation (Chenhua, Shanghai) in a homemade H-type cell (separated by Nafion 117 membrane; with magnetic stirring of 1000 rpm). The Nafion 117 was preprocessed according to the reported procedures. The Cu_x_Co_y_/Ni foams (0.5 cm^2^ × 0.5 cm^2^) were used as working electrodes, and platinum plate and Hg/HgO electrode (filled with 1 M KOH solution) were used as counter and reference electrodes, respectively. Before the testing, all the catalysts were electro-reduced at −0.2 V *vs*. RHE for 600 s in 1 M KOH solution to eliminate surface oxidation. 1 M KOH aqueous solution containing different KNO_3_^−^ concentrations (5, 10, 50, and 100 mM) were used as an electrolyte (30 ml). The electrolyte was bubbled with Ar to remove O_2_ and N_2_ for 10 min before the experiment. The electrochemical linear voltammetry (LSV) curves were obtained in a single cell. The current density was normalized to the geometric electrode area (0.25 cm^2^) unless otherwise specified. The cyclic voltammetry curves in electrochemical double-layer capacitance (C_dl_) determination were measured in a potential window where no Faradaic process occurred in an electrolyte of 1 M KOH at different scanning rates of 20, 40, 60, 80, and 100 mV s^−1^. All the potentials were converted to the RHE reference scale by *E*_(V *vs*. RHE)_ = *E*_(V *vs.* Hg/HgO)_ + 0.0591 × pH + 0.098. Note that NH_3_ volatilization in the 1 M KOH electrolytes (pH 13.6) is negligible during the 1-h electrolysis.

### Kinetic evaluation

The electrochemical kinetic analysis of NO_3_^−^RR was performed based on the Koutecký–Levich (K–L) equation, as shown in Eq. (5):

5 $$\frac{1}{{i}_{m}}=\frac{1}{{i}_{K}}+\frac{1}{0{{{{{\rm{.2nF}}}}}}{D}^{2/3}{\nu }^{-1/6}C{\omega }^{1/2}}$$

Where *i*_*m*_ is the test current; *i*_*K*_ is the kinetic current of NO_3_^−^ reduction; *n* is the number of electrons transferred in the reaction; *F* is the Faraday constant, 96485 C mol^−1^; *D* represents the effective diffusion coefficient of 0.1 mol L^−1^ NO_3_^−^ at 25 °C, 1.4 × 10^−5^ cm^2^ s^−1^; *υ* represents the kinematic viscosity of water at 25 °C, 1 × 10^−6^ m^2^ s^−1^; *C* is NO_3_^−^ concentration, mmol L^−1^；*ω* is the electrode speed, rpm.

The working electrode was prepared as follows: (1) 5 mg of catalyst powder dropped down from Cu_x_Co_y_/Ni foam and was dispersed in the solution of 600 μL isopropanol + 380 μL ultrapure water + 20 μL 5% Nafion solution, and then sonicated for at least 1 h to get a homogeneous ink; (2) 10 μL ink was drop-casted onto the rotating disk electrode (Ø, 0.196 cm2) with the loading of 0.255 mg_catalyst_ cm^−2^ for the further LSV analysis at different speeds (100, 225, 400, and 625 rpm) with a scan rate of 10 mV s^−1^. A platinum electrode and an Hg/HgO electrode were used as counter electrode and reference electrode, respectively. An aqueous solution of 1 mol L^−1^ KOH containing 100 mmol L^−1^ KNO_3_ was used as the electrolyte. Ar was used to purge the dissolved O_2_ and N_2_ from the electrolyte.

### Product detection and efficiency calculation

The NH_3_ concentration was quantified by the Nessler Reagent method^28^. The electrolytes sampled after electrolysis for 1 h were first neutralized with 0.5 M H_2_SO_4_ and then mixed with 6.25 mL ultrapure water and 0.25 mL of Nessler reagent for the chromogenic reaction. The absorbance of the mixed solution was measured at a wavelength of 420 nm after keeping it at room temperature for 30 min. The quantitation of NH_3_ was performed by the standard curves, which was built using standard NH_4_Cl solutions in 1 M KOH. The ^1^H NMR (500 MHz) determination^31^ was also carried out to quantify the ^14^NH_4_^+^ and ^15^NH_4_^+^ after electrolysis at −0.2 V *vs*. RHE for 1 h. The electrolytes were mixed with 0.4 M H_2_SO_4_ at a ratio of 500:125 to ensure adequate protonation of NH_3_. Then, 125 μl of the diluted electrolytes or standard solution were mixed with 125 μl of 10 mM maleic acid in DMSO-D6, 50 μl of 4 M H_2_SO_4_, and 300 μl of H_2_O.

The NO_3_^−^ and NO_2_^−^ in the solution were quantitatively determined by ion chromatography (IC). The possible gas products of NO_3_^−^RR, such as H_2_, N_2_, NO, NO_2_, N_2_O, and NH_3_(g) were analysed using a gas chromatography (GC) and online electrochemical mass spectrometry (OEMS)^30^.

The Faradaic efficiency was calculated according to the following equation:

6 $${{{{\rm{FE}}}}}=\frac{{{{{\rm{nc}}}}}{{{{{\rm{V}}}}}}_{{{{{\rm{catholyte}}}}}}{{{{\rm{F}}}}}}{{{{{\rm{Q}}}}}}\times 100\%$$

where *c* represents the concentration of the product, mol cm^-3^; *V*_catholyte_ is the volume of catholyte, mL; *Q* is the total amount of charge consumed, C.

The yield rate of NH_3_ was calculated according to the following equations:

7 $${{{{{\rm{Yield}}}}}}_{{{{{{\rm{NH}}}}}}_{3}}=\frac{{{{{\rm{c}}}}}{{{{{\rm{V}}}}}}_{{{{{\rm{catholyte}}}}}}}{{{{{\rm{St}}}}}}$$

8 $${{{{{\rm{Yield}}}}}}_{{{{{{\rm{mass}}}}}-{{{{\rm{NH}}}}}}_{3}}=\frac{c{V}_{{catholyte}}}{{mt}}$$

where *S* is the area of the geometrical cathode, cm^-2^; *m* is the mass of the catalyst on the cathode; *t* is the time of the electrolysis.

### Electrochemical in situ FTIR reflection analysis

Electrochemical in situ FTIR reflection spectroscopy^44^. Electrochemical thin-layer in situ FTIR spectroscopy measurements were performed on a Nicolet Nexus 8700 FTIR spectrometer equipped with a liquid N_2_-cooled system and MCT-A detector. The glassy carbon electrode loading with catalysts was used as the working electrode, which was pressed vertically on the CaF_2_ window plate to form a thin liquid layer with a thickness of about 10 μm. A platinum foil and an Hg/HgO electrode (filled with 1 M KOH solution) were used as the counter electrode and reference electrode, respectively. The incoming infrared beam was approximately aligned with the normal electrode surface. Unless otherwise noted, the sample spectra were averaged from 200 interference spectra with a resolution of 8 cm^−1^. Reference spectrum (R_Ref_) were collected at 0.4 V, and sample spectra (R_S_) were collected in the potential region from 0.4 V to −0.2 V and stepped by 100 mV. The spectra were reported as ΔR/R = (R_S_ − R_Ref_)/ R_Ref_.

Attenuated Total Reflection in situ FTIR reflection spectroscopy^36^. The gold-plated Si prism with catalysts were assembled into a homemade spectral-electrochemical cell, which contained a carbon sheet as a counter electrode and Hg/HgO electrode as a reference electrode. It was then fixed in a homemade optics system built in the chamber of a Nicolet Nexus 8700 FTIR spectrometer for electrochemical ATR-FTIR measurements at an incidence angle of *ca*. 65°. The ATR-FTIR spectra were reported in the same way of thin-layer in situ FTIR, except that R_Ref_ was taken at 0.5 V and R_S_ were collected in the potential region from 0.5 to −0.4 V.

### SHINERS analysis

SHINERS spectra were recorded in a custom-made in situ Raman spectroelectrolysis cell using an XplorA confocal microprobe Raman spectrometer (HORIBA Jobin Yvon)^63^. The excitation wavelength of the laser was 637.8 nm and came from a He-Ne laser with a power of about 6 mW. The electrochemically polished gold electrode (diameter 3 mm) was modified by 10 μL catalyst ink with 10 μL homemade shell-isolated gold nanoparticle, which was provided by Prof. Jian-Feng Li at Xiamen University, China, and applied as the working electrode. Hg/HgO electrode was used as the reference electrode, and platinum wires was used as the counter electrode. A long-focus objective (8 mm) of A × 50 magnification was used. A Si wafer (520.6 cm^−1^) was used to calibrate the Raman frequency before the experiment. The SHINERS spectra were obtained using the cumulative results of four tests for 30 s each.

### EPR Experiments

5,5-dimethyl-1-pyrroline *N*-oxide (DMPO) was used to capture the instable hydrogen radical to form the DMPO-H adduct to generate EPR spectra^64^. In the experiments, 5 ml electrolyte was mixed with 10 μL DMPO and was deoxygenated by bubbling Ar. The potentiostatic electrolysis was carried out for 5 min in the H-type cell under the protection of Ar. EPR measurement was performed by Bruker EMX-10/12 spectrometer operating at a frequency near 9.5 GHz, sweep width of 200 G and power of 20 mW.

### DFT calculations

All DFT calculations in this work were carried out with the Vienna Ab initio Simulation Package (VASP)^65^. And the projector augmented-wave (PAW) pseudopotential was selected to deal with the core-valence interaction^66^. The generalized gradient approximation (GGA) of Perdew–Burke–Ernzerhof (PBE) was used to account for the exchange and correlation of electronics and the cut-off energy of plane-wave was 600 eV^67^. The energy convergence criterion was within 10^-5^ eV and the Hellmann–Feynman force was smaller than 0.01 eV Å^−1^ on each atom. The converged unit cell models of Cu (3.64 × 3.64 × 3.64 Å^3^), Co (3.52 × 3.52 × 3.52 Å^3^), and CuCo (3.78 × 3.49 × 3.49 Å^3^) were used in DFT calculations, respectively. The dimension of a 2 × 2 supercell of Cu (111) (8.91 × 10.28 Å^2^), a 2 × 2 supercell of Co (111) (8.62 × 9.95 Å^2^) and a 2 × 2 supercell of CuCo (111) (9.03 × 9.87 Å^2^) were used, respectively. These supercells were constructed and contained three layers and a sufficient vacuum layer of 15 Å thicknesses. For the structural optimization, the bottom two layers were fixed and the top layer was fully relaxed^68^. For unit cell geometry optimization, an 8 × 8 × 8 k-point analysis was used. A grid of 3 × 3 × 1 k-point mesh was used for these supercell calculations^15^. The calculations of all molecules and intermediate species on Co(111) and CuCo(111) were performed with spin polarization^69^. Dipole corrections in the z direction were included in all computations to minimize inaccuracies in the total energy because of simulated slab interactions. The spin polarization was not taken into account in the calculations of intermediate species on Cu(111) due to the spin polarization did not affect the Cu (111) calculations.

## Supplementary information

Supplementary Information

Peer Review File

## Data Availability

The raw data of the figures in the main manuscript are available in figshare with the identifier(s) 10.6084/m9.figshare.21671075. All other data needed to evaluate the conclusions in the paper are present in the paper and the Supplementary Information or can be obtained from the corresponding authors on reasonable request.

## Code availability

The code used in this work can be obtained from the corresponding authors on reasonable request.
