# Supplementary material for: Ampere-level current density ammonia electrochemical synthesis using CuCo nanosheets simulating nitrite reductase bifunctional nature
Source: Nat Commun. 2022 Dec 22;13:7899. doi: 10.1038/s41467-022-35533-6 (PMC9780304; doi:10.1038/s41467-022-35533-6)
Supplement: Supplementary file 1 — Supplementary Information [file 41467_2022_35533_MOESM1_ESM.pdf]

Supplementary Information

Ampere-Level Current Density Ammonia Electrochemical Synthesis using CuCo nanosheets simulating Nitrite reductase bifunctional Nature

Jia-Yi Fang[a,†], Qi-Zheng Zheng[a,†], Yao-Yin Lou [a,b,*] , Kuang-Min Zhao[a], Sheng-Nan Hu[a], Guang Li[a], Ouardia Akdim[c], Xiao-Yang Huang[a,c], and Shi-Gang Sun [a,*]

Affiliations

[a] State Key Laboratory of Physical Chemistry of Solid Surfaces, Department of Chemistry, College of Chemistry and Chemical Engineering, Xiamen University, Xiamen, 361005, China.

[b] School of Chemical and Environmental Engineering, College of Chemistry, Chemical Engineering and Materials Science, Soochow University, Suzhou, Jiangsu 215123, China.

[c] Cardiff Catalysis Institute, School of Chemistry, Cardiff University, Main Building, Park Place, Cardiff, CF10 3AT, UK.

[†]These authors contributed equally to this work.

*Corresponding authors. Email: yylou@suda.edu.cn (Yao-Yin Lou); sgsun@xmu.edu.cn (Shi-Gang Sun).

1. Supplementary Figures. **4**

Supplementary Fig. S1. XRD spectra of catalysts with different Cu-to-Co ratios.

Supplementary Fig. S2. HRTEM images of Cu and Co catalysts.

Supplementary Fig. S3. SEM images of catalysts with different Cu-to-Co ratios.

Supplementary Fig. S4. XPS peak spectra of catalysts with different Cu-to-Co ratio.

Supplementary Fig. S5. XAS spectra of $Cu_{50}Co_{50}$ catalysts.

Supplementary Fig. S6. Bader analysis for Cu(111), Co(111) and CuCo(111).

Supplementary Fig. S7. 2D atomic electron density differences of CuCo(111).

Supplementary Fig. S8. *j-E* plots on Ni foam and $Cu_{50}Co_{50}$ / Ni foam.

Supplementary Fig. S9. NO_3^- removal rate on Ni foam and $Cu_{50}Co_{50}$ / Ni foam.

Supplementary Fig. S10. *j-E* plots and the Koutecký-Levich analysts of different catalysts.

Supplementary Fig. S11. Electron transfer numbers of NO_3^-RR on Cu and Co catalysts.

Supplementary Fig. S12. *j-E* plots at different NO_3^- concentration and the corresponding lg(-*j*)-lgC plots.

Supplementary Fig. S13. EIS of Cu, Co and $Co_{50}Cu_{50}$.

Supplementary Fig. S14. H-type electrolytic cell's photo.

Supplementary Fig. S15. The quantification of NH_3 NO_2^- and NO_3^-.

Supplementary Fig. S16. The OEMS results for the possible gaseous products of NO_3^-RR.

Supplementary Fig. S17. ECSA measurements of catalysts with different Cu-to-Co ratio.

Supplementary Fig. S18. ECSA measurements of Ni foam.

Supplementary Fig. S19. Bias-current density for NH_3 and NH_3 production yield on catalysts with different Cu/Co ratio.

Supplementary Fig. S20. Current density and yield rate for NH_3 on $Cu_{50}Co_{50}$/Ni foam.

Supplementary Fig. S21. ^{1}H NMR analysis for the quantitative of ammonia.

Supplementary Fig. S22. The time-dependent current density curves on $Cu_{50}Co_{50}$ modified Ni foam.

Supplementary Fig. S23. FE of H_2, NO_2^- and NH_3 for $Cu_{50}Co_{50}$ catalyst at different electrode potentials.

Supplementary Fig. S24. The current density at different electrode potentials.

Supplementary Fig. S25. FE_{NH_3} and product yield rate for NH_3 on $Cu_{50}Co_{50}$/Ni foam, $Cu_{50}Co_{50}$/Cu foam, $Cu_{50}Co_{50}$/CP and Co/ Cu foam.

Supplementary Fig. S26. The characteristic of $Cu_{50}Ni_{50}$ catalyst after NO_3^-RR operation.

Supplementary Fig. S27. FE_{NH_3} of $Cu_{50}Co_{50}$ catalyst in different initial NO_3^- concentrations.

Supplementary Fig. S28. The electrochemical performance on Cu, $Cu_{50}Co_{50}$ and Co catalysts in neutral electrolyte.

Supplementary Fig. S29. Electrochemical *in situ* ATR-FTIR spectra of NO_3^-RR in D_2O.

Supplementary Fig. S30. Electrochemical *in situ* ATR-FTIR spectra of NO_3^-RR.

Supplementary Fig. S31. EPR spectra of DMPO spin-trapping H*.

Supplementary Fig. S32. Electrochemical SHINERS spectra of NO_3^-RR on Au@SiO_2.

Supplementary Fig. S33. Electrochemical SHINERS spectra of NO_3^-RR on Cu and Co catalysts.

Supplementary Fig. S34. The thermodynamic cycle used to calculate the Gibbs free energy of NO_3^- in the aqueous phase ($NO^-_{3(l)}$) adsorbed onto the electrode surface.

2. **Supplementary Tables.** **22**

Supplementary Table S1. Summary of ICP-OES analysis results for different Cu/Co ratio.

Supplementary Table S2. Comparison of the electrocatalytic NO_3^-RR performances of $Cu_{50}Co_{50}$ catalysts with other extensively reported electrocatalysts.

Supplementary Table S3. TIR peaks for the *in situ* NO_3^-RR on $Cu_{50}Co_{50}$, Cu and Co catalysts.

Supplementary Table S4. Raman scattering peaks for *in situ* nitrates reduction on $Cu_{50}Co_{50}$, Cu and Co catalysts.

Supplementary Table S5. The electronic energies (E), zero-point energies (ZPE) and entropy (TS) corrections for $H_2O_{(l)}$, $H_{2(g)}$, $NH_{3(g)}$ and $HNO_{3(g)}$ used in the Gibbs free energy calculations.

Supplementary Table S6. Adsorption configurations of adsorption species on Cu(111), Co(111) and CuCo(111) surfaces.

Supplementary Table S7. The correction of zero-point energy (ZPE) of adsorption species on different catalysts' surfaces.

Supplementary Table S8. The correction of entropy (TS) of adsorption species on different catalysts' surfaces.

Supplementary Table S9. Calculated electronic energies (E) of adsorption species on different catalysts' surfaces.

Supplementary Table S10. Calculated Gibbs free energies (ΔG) of adsorption species for NO_3^-RR on different catalysts' surfaces with respect to the reference $NO_3^-{}_{(l)}$ + *.

Supplementary Table S11. Calculated Gibbs free energies (ΔG) of adsorption species for HER on different catalysts' surfaces with respect to the reference of $H^+ + e^- + *$.

Supplementary Table S12. top view and side view of Cu(111), Co(111) and CuCo(111) with different surface exposure structures and the corresponding adsorption configuration of $*NO_3$ and $*NO_2$.

Supplementary Table S13. Calculated Gibbs free energies (in eV) of $*NO_3$ and $*NO_2$ on Cu(111), Co(111) and CuCo(111) with different surface exposure structures at 0 V *vs.* RHE.

Supplementary Table S14. Calculated Gibbs free energies (in eV) of $*NO_3$ and $*NO_2$ on Cu(111), Co(111) and CuCo(111) with different surface exposure structures at -0.2 V *vs.* RHE.

3. Supplementary Notes. **33**

Supplementary Note S1. Details of DFT calculations.

Supplementary Note S2. The reference used in the Gibbs free energy calculations.

Supplementary Note S3. The initial date calculated for the Gibbs free energy calculations.

Supplementary Note S4. Stable configurations of the intermediates on different CuCo surface models.

4. References for Supplementary Information. **36**

Supplementary Figures and Tables.

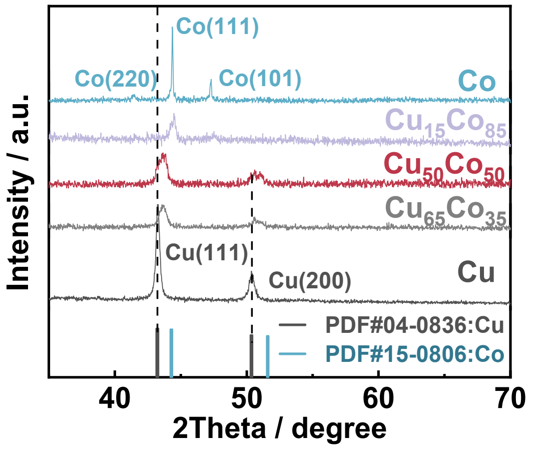

Supplementary Fig. S1. XRD spectra of catalysts with different Cu-to-Co ratios.

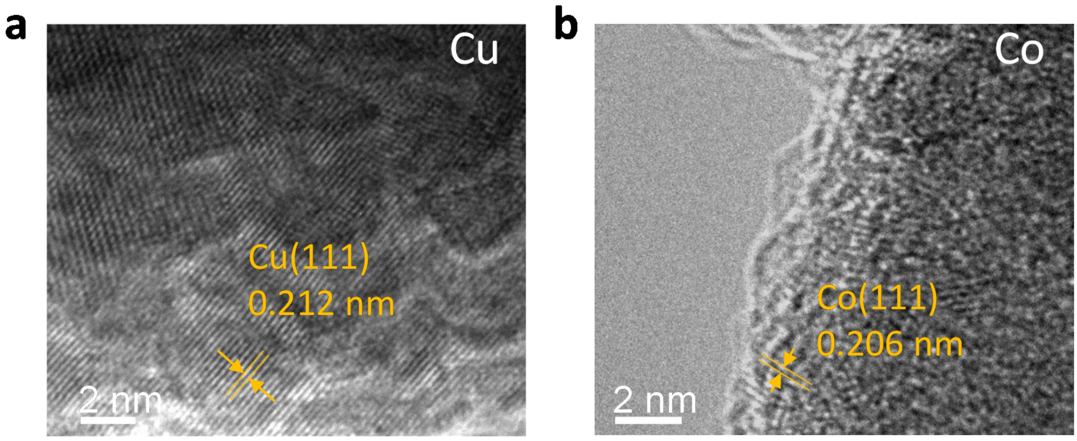

Supplementary Fig. S2. HRTEM images of Cu (a) and Co (b) catalysts.

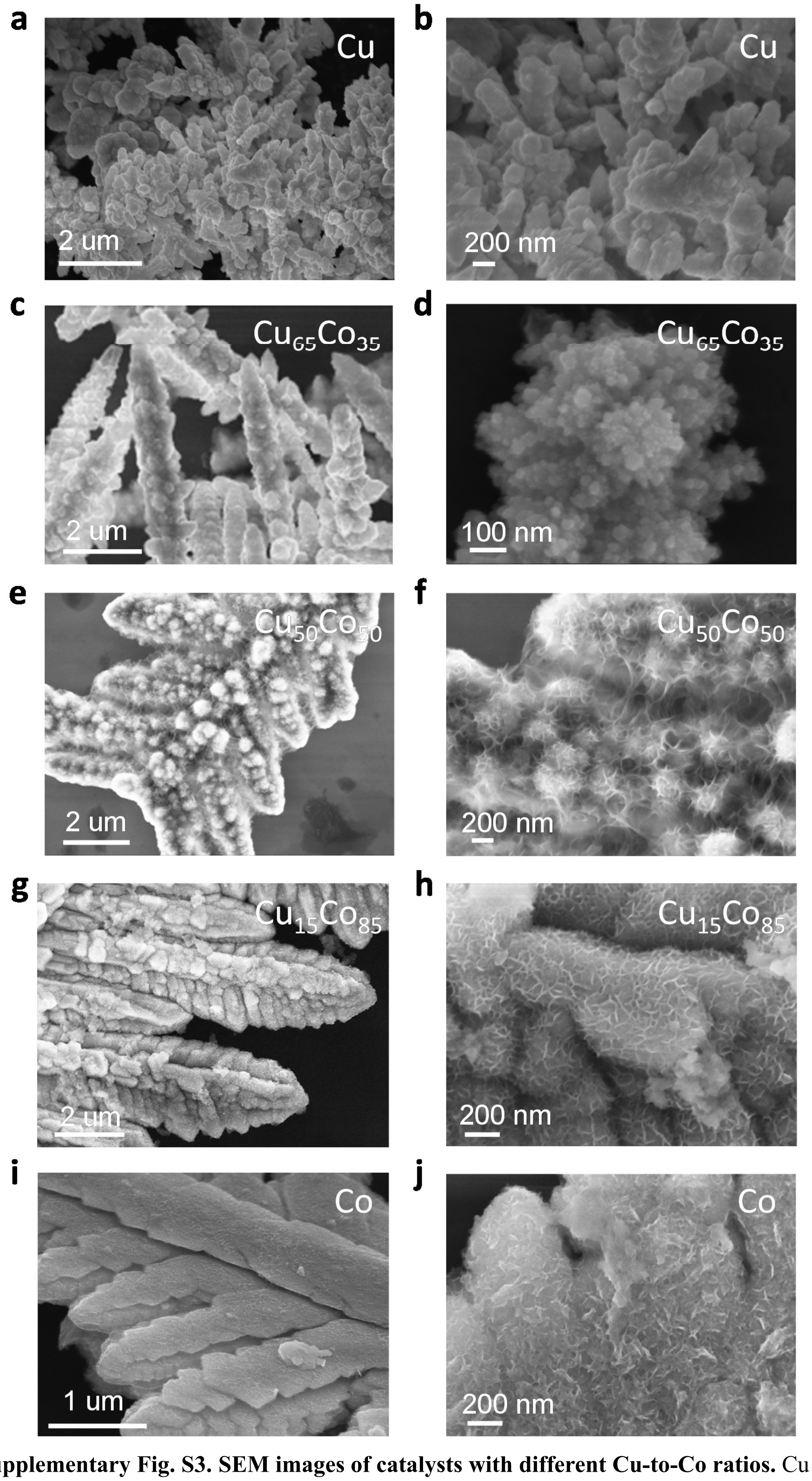

Supplementary Fig. S3. SEM images of catalysts with different Cu-to-Co ratios. Cu (**a**) (**b**), $Cu_{65}Co_{35}$ (**c**) (**d**), $Cu_{50}Co_{50}$ (**e**) (**f**), $Cu_{15}Co_{85}$ (**g**) (**h**), Co (**i**) (**j**).

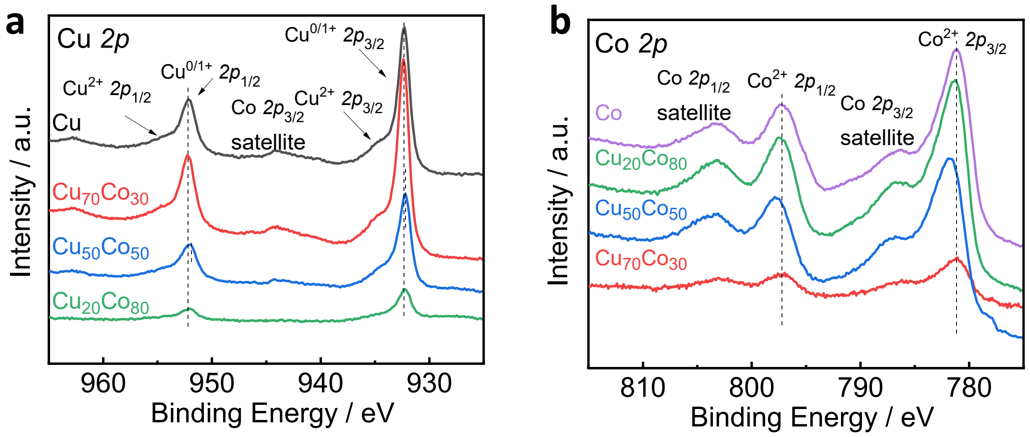

Supplementary Fig. S4. XPS peak spectra of catalysts with different Cu-to-Co ratios. Cu 2*p* (**a**) and Co 2*p* (**b**)

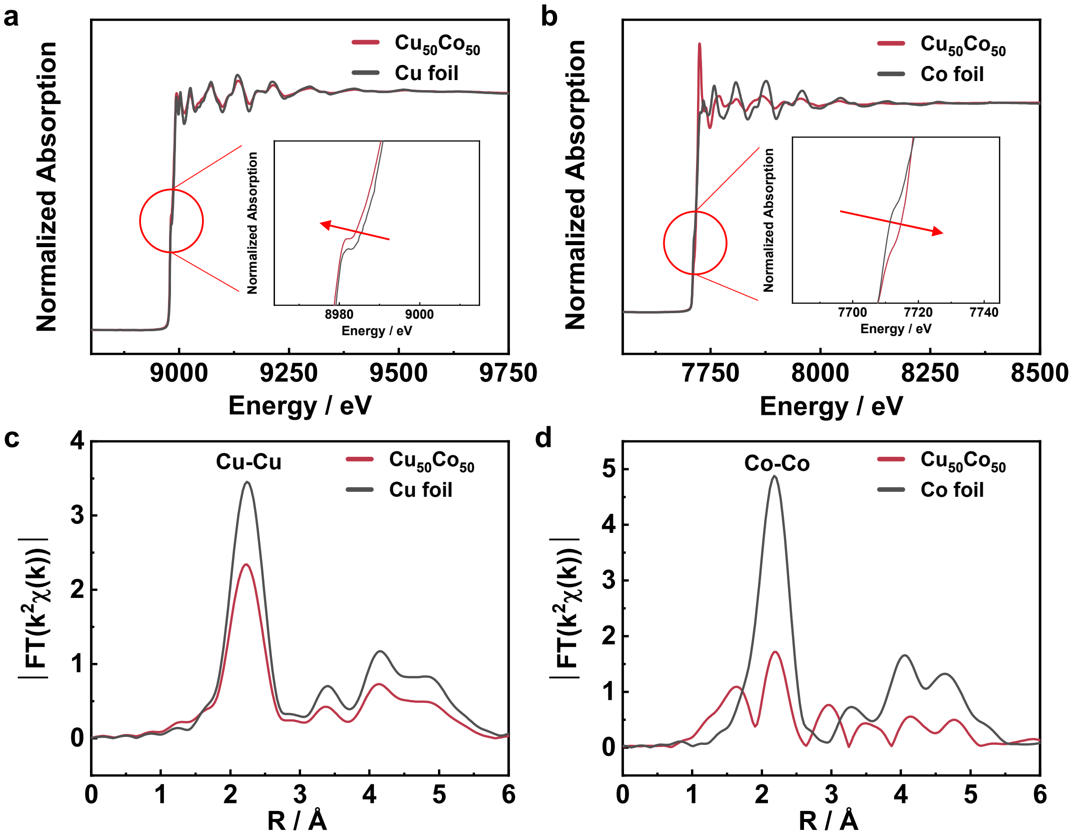

Supplementary Fig. S5. XAS analyst of $Cu_{50}Co_{50}$ catalyst. Extended X-ray absorption fine structure (XANES) spectra of Cu (a) and Co (b) K-edge of $Cu_{50}Co_{50}$, compared to the metallic Cu and Co foil used as references. Cu (c) and Co (d) K-edge FT-EXAFS spectra.

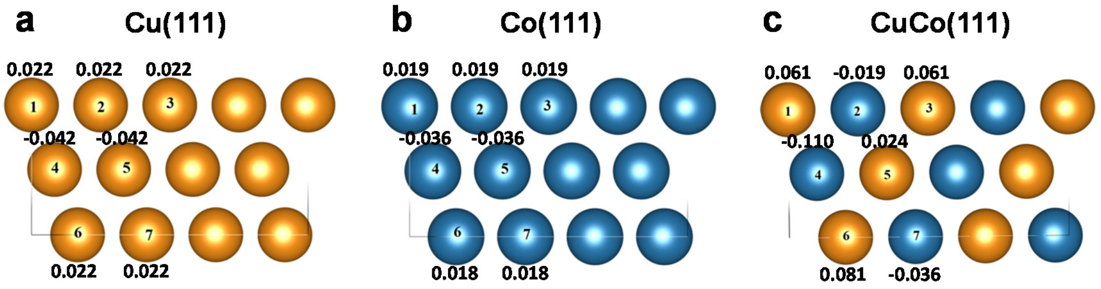

Supplementary Fig. S6. Bader analysis for Cu(111), Co(111) and CuCo(111).

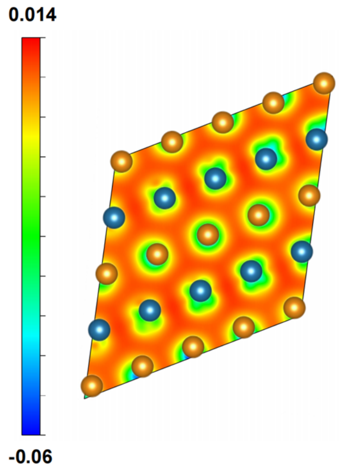

Supplementary Fig. S7. 2D atomic electron density differences of CuCo(111).

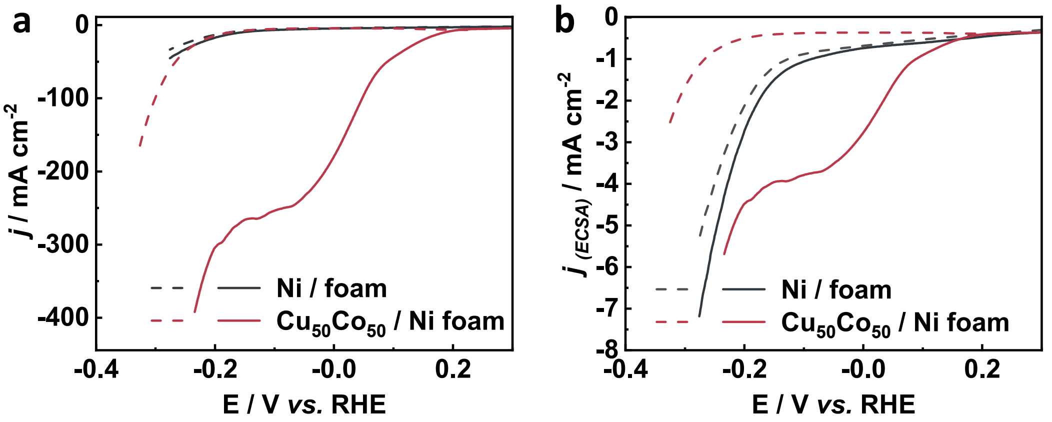

Supplementary Fig. S8. ***j-E*** **plots on Ni foam and** $\mathbf{Cu_{50}Co_{50}}$ **/ Ni foam.** Current density normalized by geometric area (**a**) or electrochemical active surface area ($j_{(ECSA)}$) (**b**)-Electrode potential (*E*) curve (80% i*R* corrected) on Ni foam and $Cu_{50}Co_{50}$ / Ni foam in 1 M KOH solution containing 100 mM KNO_3 (solid lines) or in the absence of KNO_3 (dotted line) at a sweep rate of 1 mV s^{-1}.

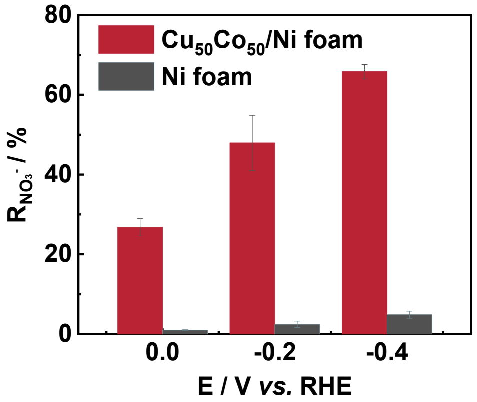

Supplementary Fig. S9. NO_3^- removal rate on $Cu_{50}Co_{50}$ / Ni foam and Ni foam. The results were performed in electrolyte of 100 mM KNO_3 + 1 M KOH.

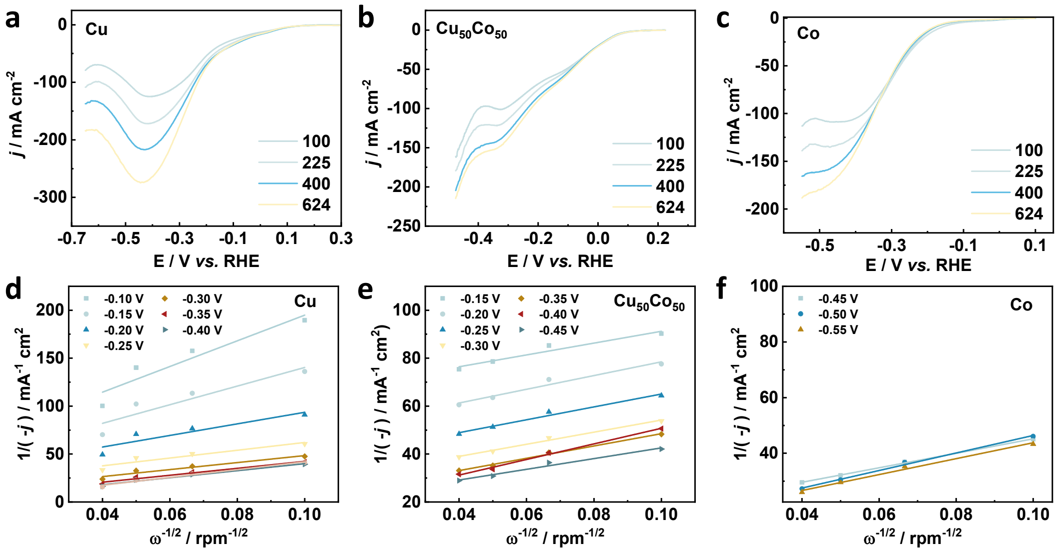

Supplementary Fig. S10. *j*-*E* plots at different rotation rates in 100 mM KNO_3 + 1 M KOH on Cu **(a)**, $Cu_{50}Co_{50}$ **(b)** and Co **(c)**, and the corresponding Koutecký-Levich curves of different electrode potentials on Cu (**d**); $Cu_{50}Co_{50}$ (**e**) and Co (**f**).

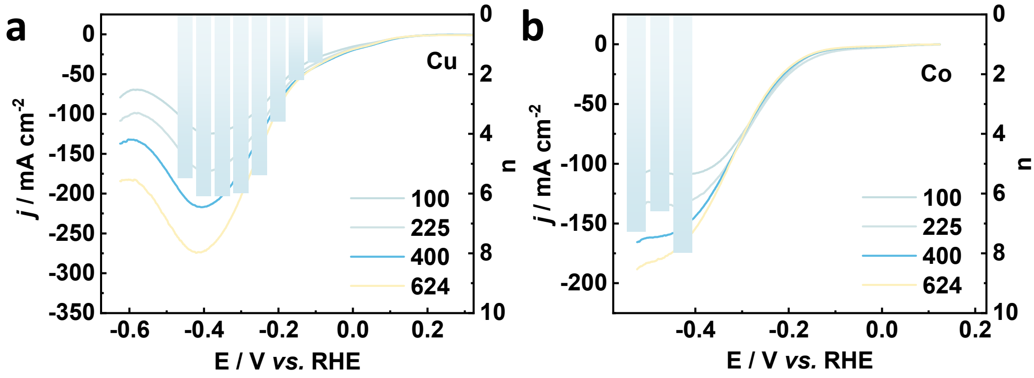

Supplementary Fig. S11. Electron transfer numbers of NO_3^-RR on Cu and Co catalysts. The results were calculated by the K-L equation for Cu (**a**) and Co (**b**), in 100 mM KNO_3 + 1 M KOH.

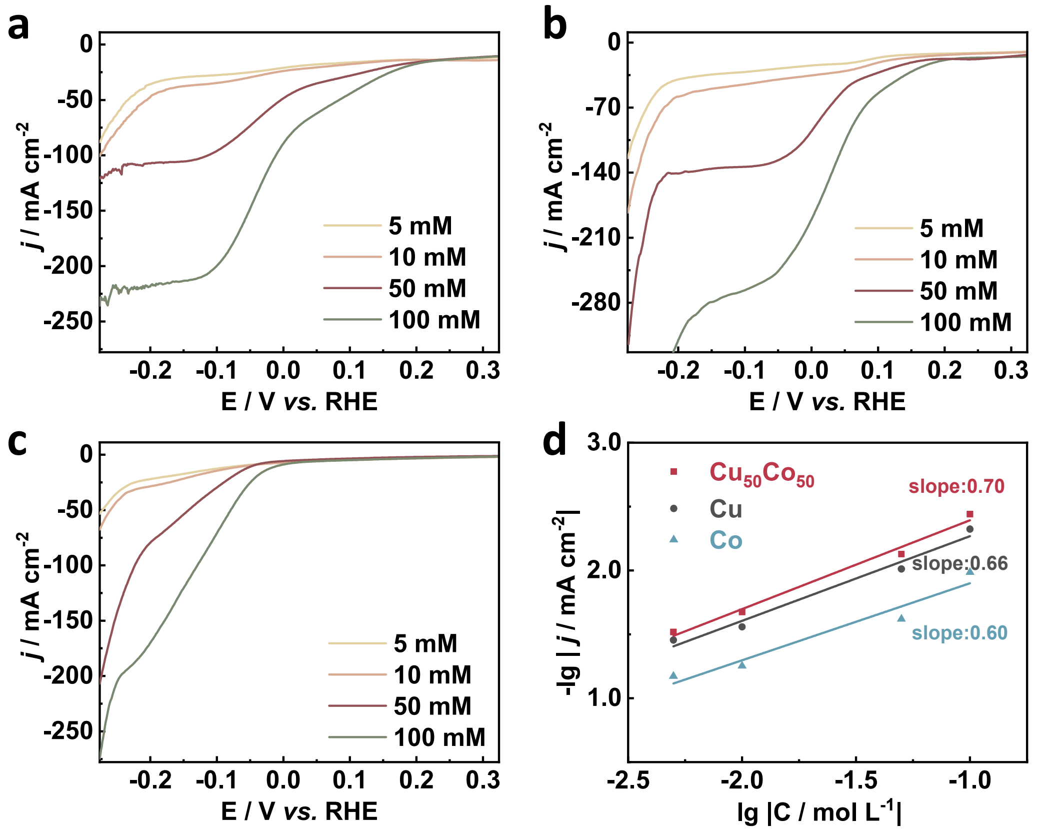

Supplementary Fig. S12. *j-E* plots at different NO_3^- concentration and the corresponding lg(-*j*)-lgC plots. The curves were got in 1M KOH containing of 5 mM, 10 mM, 50mM or 100 mM KNO_3 at 1 mV s^{-1} on Cu (**a**), $Cu_{50}Co_{50}$ (**b**) and Co (**c**) catalysts. lg(-*j*)-lgC plots for different catalysts were calculated at -0.1 V *vs.*RHE (**d**).

By calculating the relationship between the current density and the nitrate concentration, the rate of NO_3^-RR and the NO_3^- concentration formed a quasi-first-order reaction relationship. Combined with previous analysis of the Tafel slope results, the rate-determinate step (RDS) was the first electron transfer where adsorbed NO_3^- species were reduced. Prior to this step, we speculated that the nitrates were simultaneously adsorbed and desorbed at the catalyst surface, reaching an equilibrium state to satisfy a quasi-first-order reaction relationship with nitrates' concentration.

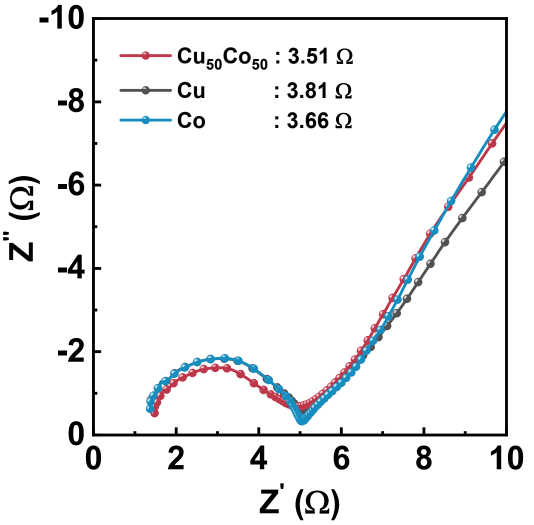

Supplementary Fig. S13. EIS of Cu, Co and $Co_{50}Cu_{50}$ in 1 M KOH.

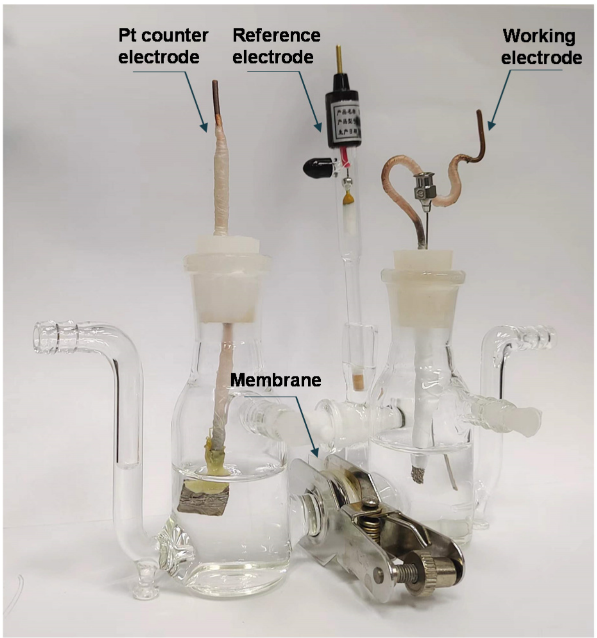

Supplementary Fig. S14. H-type electrolytic cell's photo.

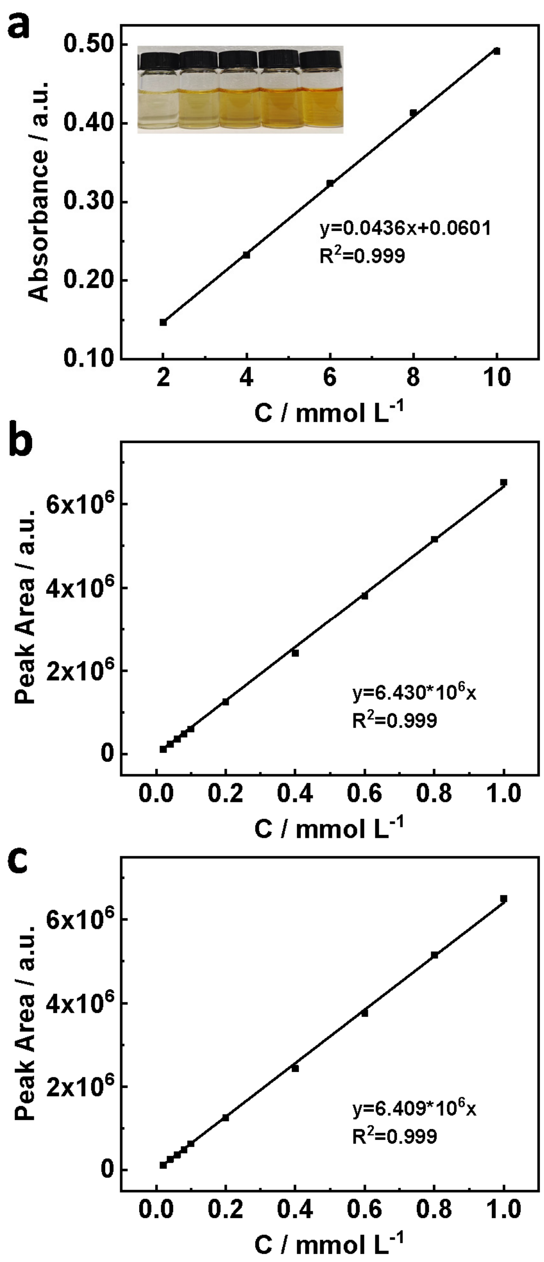

Supplementary Fig. S15. The quantification of NH_3 NO_2^- and NO_3^-. (**a**) The linear standard curve for the calculation of NH_3 production using Nessler Reagent method. The linear standard curve for the calculation of NO_2^- (**b**) and NO_3^- (**c**) using ion chromatography.

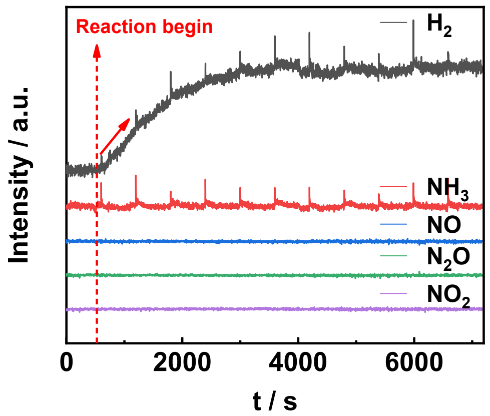

Supplementary Fig. S16. The OEMS result for the possible gaseous products of NO_3^-RR. (H_2, N_2, NO, NO_2, N_2O and NH_3(g))

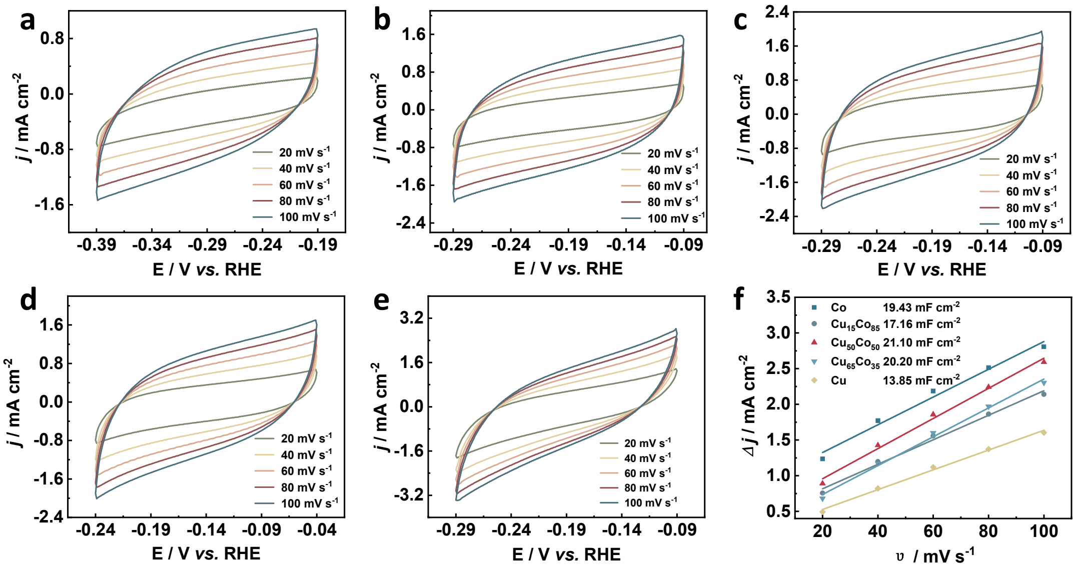

Supplementary Fig. S17. ECSA measurements of the catalysts with different Cu-to-Co ratio. The cyclic voltammetry curves were obtained on Cu (**a**), $Cu_{65}Co_{35}$ (**b**), $Cu_{50}Co_{50}$ (**c**) $Cu_{15}Co_{85}$ (**d**) and Co (**e**) catalysts in 1 M KOH. Double layer capacitance for each catalyst (Reference Cu foil: 29μF cm^{-2}) [1] (**f**).

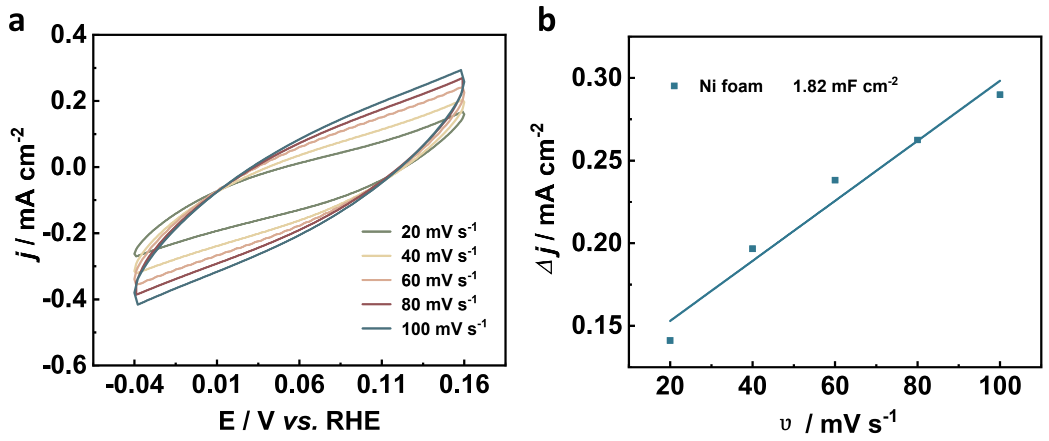

Supplementary Fig. S18. ECSA measurements of Ni foam (**a**) and corresponding double layer capacitance (**b**).

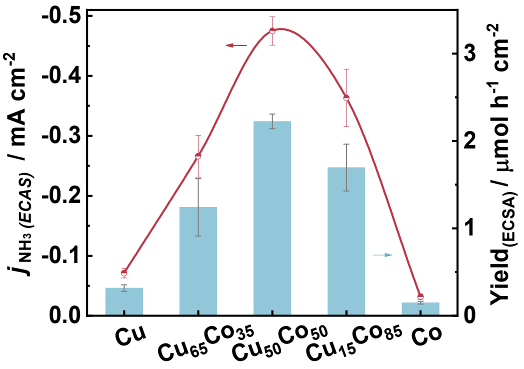

Supplementary Fig. S19. Bias-current density for NH_3 and NH_3 production yield normalized by ECSA for catalysts with different Cu/Co ratio in 100 mM KNO_3 + 1M KOH.

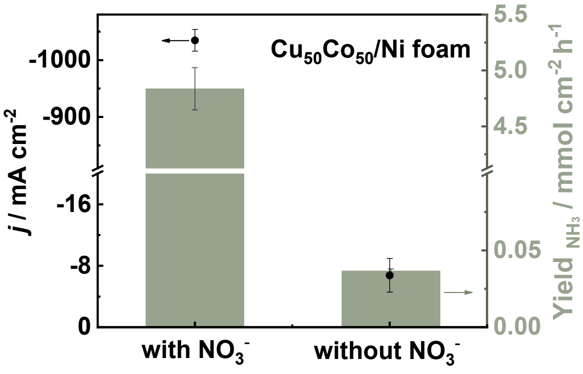

Supplementary Fig. S20. Current density and yield rate for NH_3 on $Cu_{50}Co_{50}$/Ni foam at -0.2 V *vs.* RHE in 1 M KOH electrolyte with or without 100 mM KNO_3.

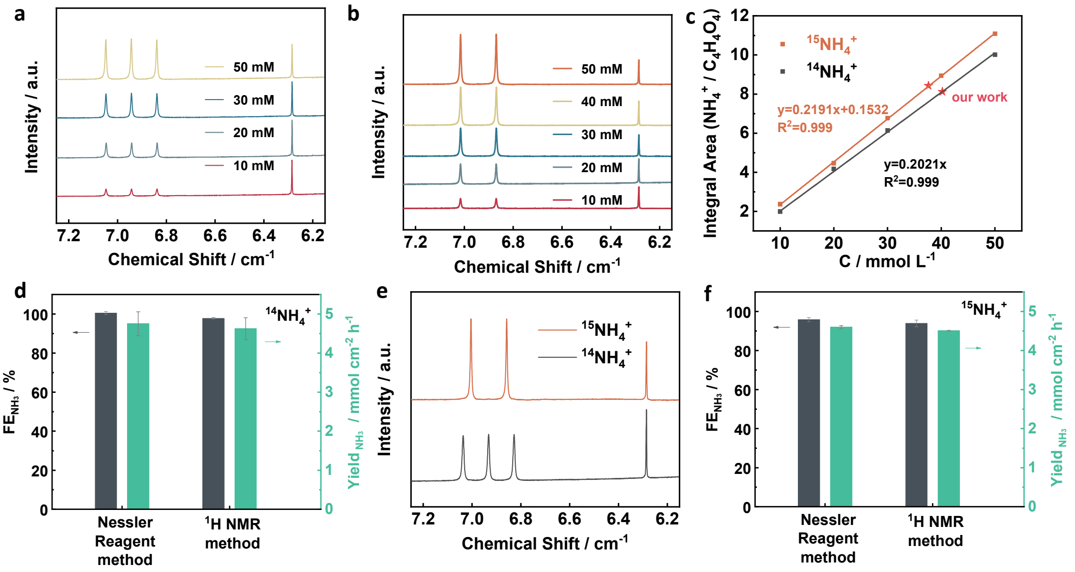

Supplementary Fig. S21. ^{1}H NMR analysis for the quantitative of ammonia. ^{1}H NMR spectra of different concentrations of $^{14}NH_4Cl$ (**a**) and $^{15}NH_4Cl$ (**b**) standard solution and the calibration curves of normalized integral area between NH_4^+ and $C_4H_4O_4$ *vs.* the concentration of NH_4^+ (**c**). **d** The $^{14}NH_3$ yield rate and Faradaic efficiency detected by ^{1}H NMR spectroscopy and the Nessler reagent method at -0.2 V vs. RHE. **e** ^{1}H NMR spectrum of the electrolyte after the electrolysis of $^{14}NO_3^-$ and $^{15}NO_3^-$ at -0.2 V vs. RHE. **f** The $^{15}NH_3$ yield rate and Faradaic efficiency detected by ^{1}H NMR spectroscopy and the Nessler reagent method at -0.2 V vs. RHE.

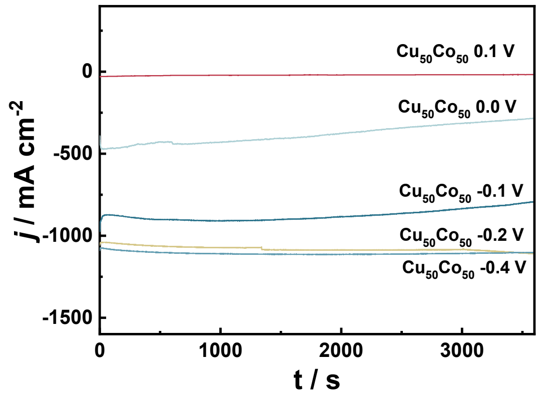

Supplementary Fig. S22. The time-dependent current density curves on $Cu_{50}Co_{50}$ modified Ni foam. The curves were got at different electrode potential in 1 M KOH solution containing 100 mM KNO_3 and with magnetic stirring speed of 1000 rpm.

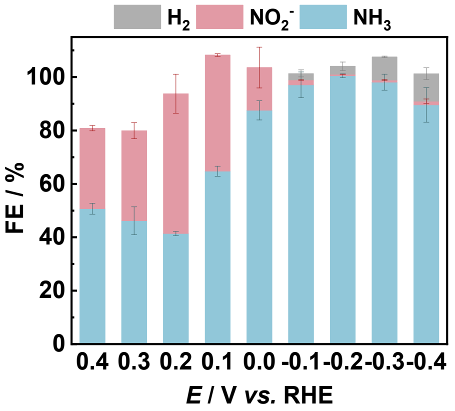

Supplementary Fig. S23. ***FE*** **of H_2, NO_2^- and NH_3 for $Cu_{50}Co_{50}$ catalyst at different electrode potentials.** Electrolyte of 100 mM KNO_3 + 1M KOH.

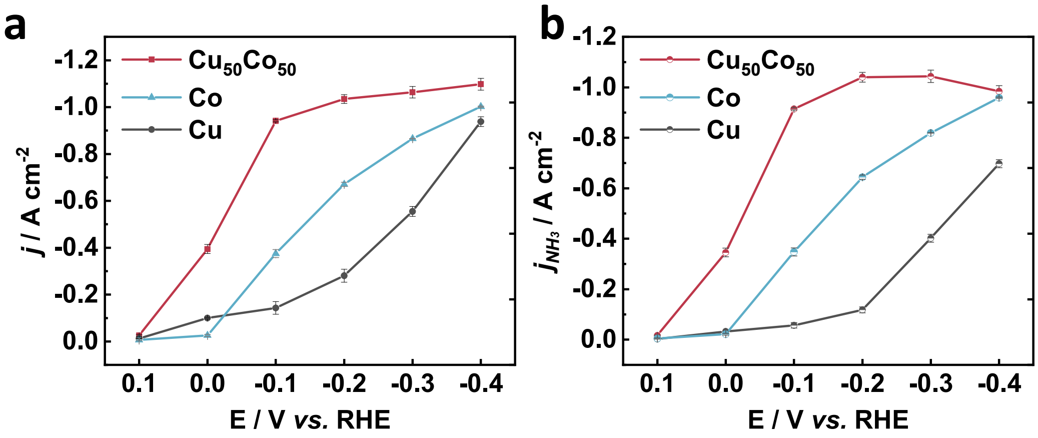

Supplementary Fig. S24. The current density at different electrode potentials. NO_3^-RR current density (**a**) and bias-current density for NH_3 (**b**) on $Cu_{50}Co_{50}$, Cu and Co in 100 mM KNO_3 +1M KOH.

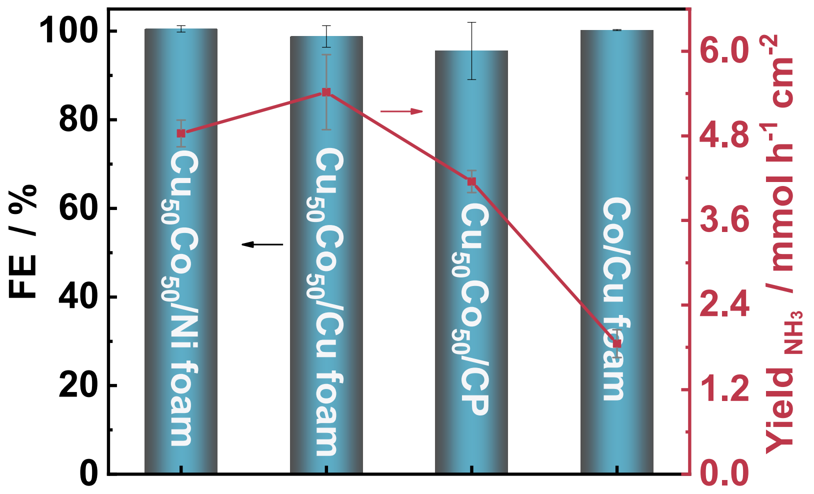

Supplementary Fig. S25. FE_{NH_3} and product yield rate for NH_3 on $Cu_{50}Co_{50}$/Ni foam, $Cu_{50}Co_{50}$/Cu foam, $Cu_{50}Co_{50}$/CP and Co/ Cu foam at -0.2 V *vs.* RHE in 100 mM KNO_3 + 1 M KOH electrolyte.

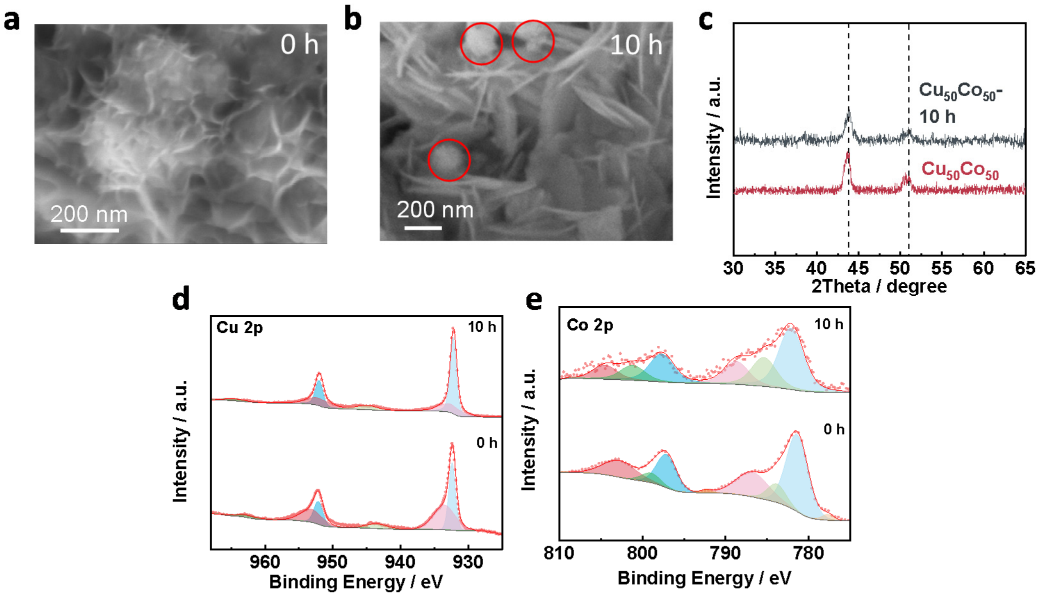

Supplementary Fig. S26. The characteristic of $Cu_{50}Ni_{50}$ catalyst after NO_3^-RR operation. SEM images of the $Cu_{50}Ni_{50}$ catalyst before (**a**) and after 10 periods of 1 h (**b**) electrocatalytic NO_3^-RR. XRD spectra (**c**) and XPS peaks spectra of Cu 2*p* (**d**) and Co 2*p* (**e**) of $Cu_{50}Co_{50}$ catalyst before and after 10 periods of 1 h NO_3^-RR operation at -0.2 V *vs.* RHE.

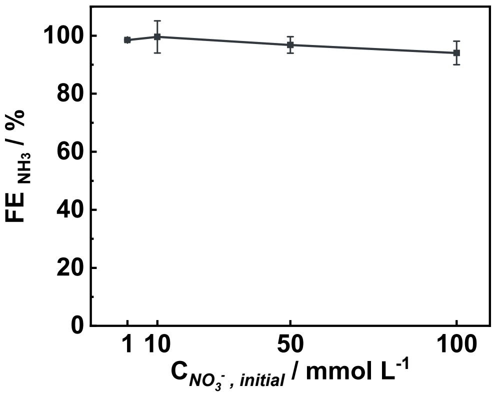

Supplementary Fig. S27. FE_{NH_3} of $Cu_{50}Co_{50}$ catalyst in different initial NO_3^- concentrations. The experiment were performed at an electrode potential of -0.1 V *vs.* RHE in 1 M KOH solutions with initial NO_3^- concentrations of 1, 10, 50, 100 mM.

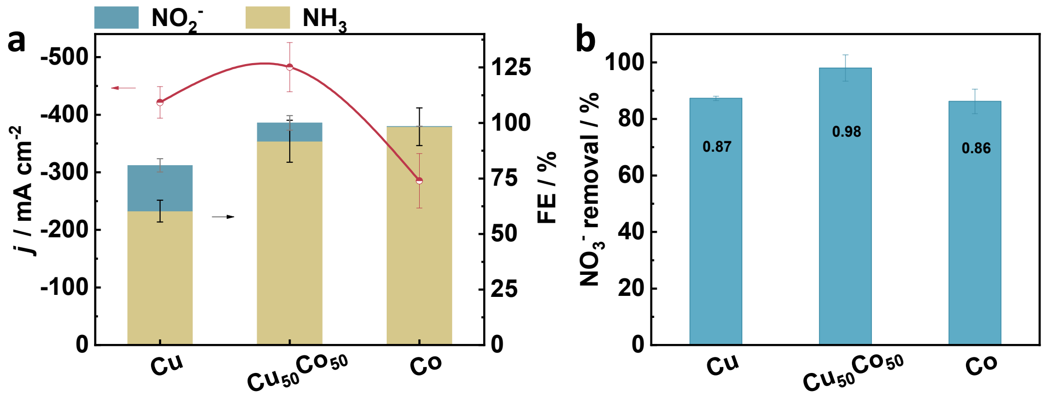

Supplementary Fig. S28. The electrochemical performance on Cu, $Cu_{50}Co_{50}$ and Co catalysts in neutral electrolyte. Current density and FE_{NH_3} and $FE_{NO_2^-}$ of NO_3^-RR in 100 mM KNO_3 + 0.5 M K_2SO_4 neutral electrolyte (**a**) and NO_3^- removal rate after 2 hour reduction in 0.1 M K_2SO_4 electrolyte with 200 mg NO_3^- (**b**) on Cu, $Cu_{50}Co_{50}$ and Co catalysts at -0.2 V *vs.* RHE.

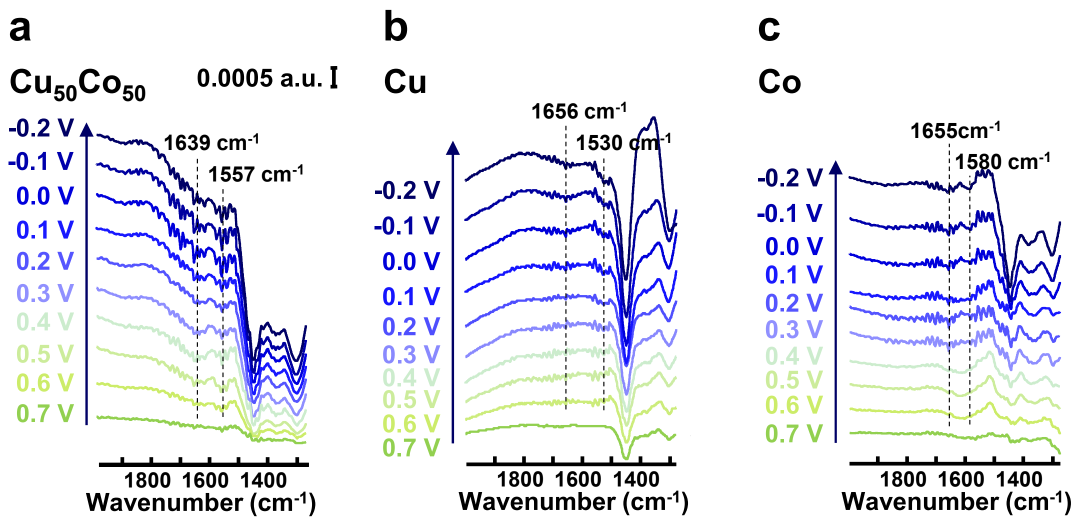

Supplementary Fig. S29. Electrochemical *in situ* ATR-FTIR spectra of NO_3^-RR in D_2O. Spectra on $Cu_{50}Co_{50}$ (**a**), Cu (**b**) and Co (**c**) in electrolyte of 100 mM KNO_3 + 1 M KOH with D_2O as the solvent.

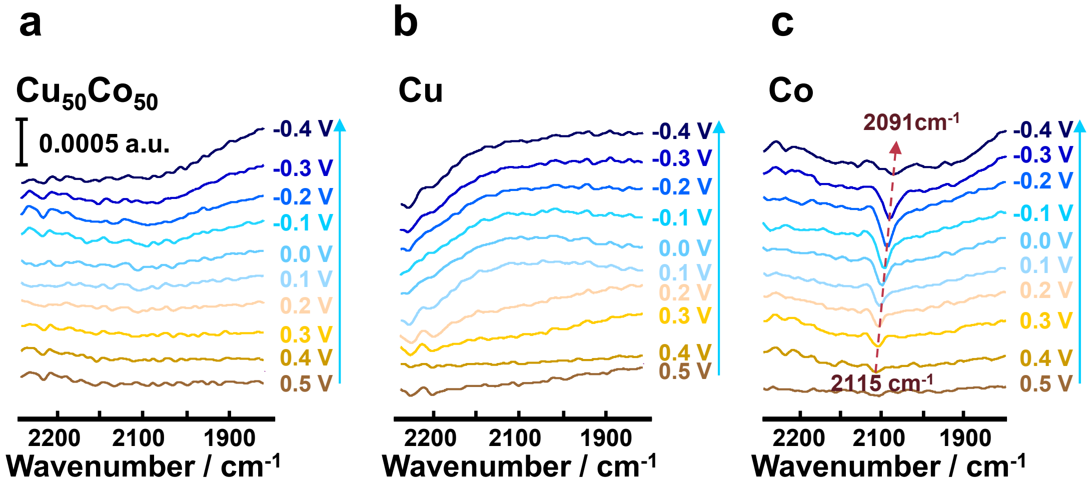

Supplementary Fig. S30. Electrochemical *in situ* ATR-FTIR spectra of NO_3^-RR. The spectra on $Cu_{50}Co_{50}$ (**a**), Cu (**b**) and Co (**c**) catalysts in 100 mM KNO_3 +1M KOH.

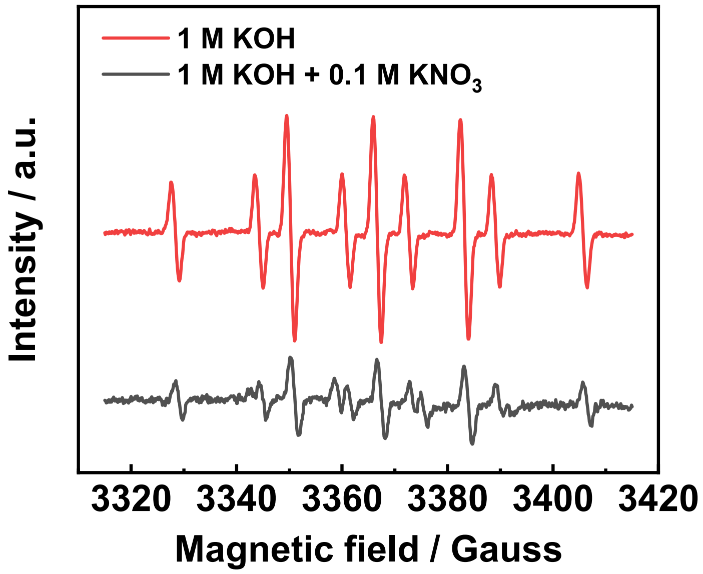

Supplementary Fig. S31. EPR spectra of DMPO spin-trapping H*. The DMPO-H* stemmed from $Cu_{50}Co_{50}$ electrodes at -0.4 V *vs.* RHE in 1 M KOH solution with or without NO_3^-, respectively.

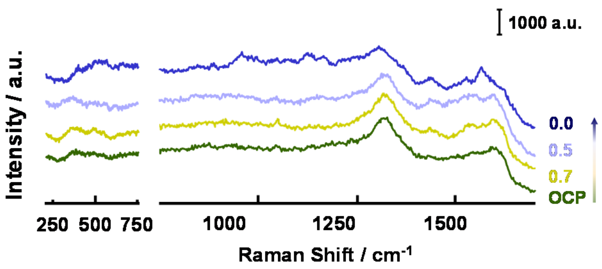

Supplementary Fig. S32. Electrochemical SHINERS spectra of NO_3^-RR on Au@SiO$_2$. Spectra between 230-750 cm^{-1} and 750-1700 cm^{-1} in 100 mM KNO_3 + 10 mM KOH.

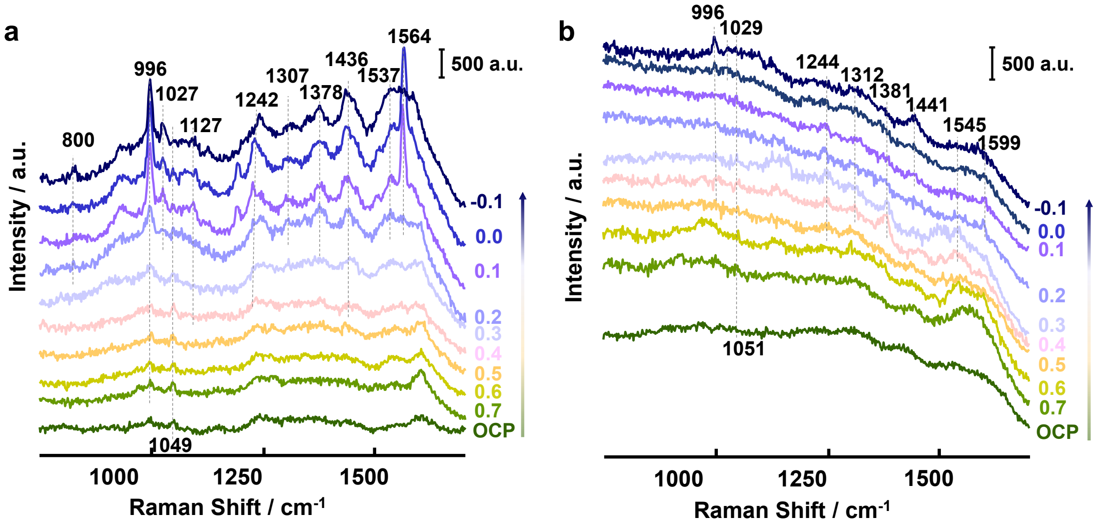

Supplementary Fig. S33. Electrochemical SHINERS spectra of NO_3^-RR on Cu and Co catalysts. Spectra between 750-1700 cm^{-1} on Cu (**a**) and Co (**b**) in 100 mM KNO_3 + 10 mM KOH during cathodic polarization from 0.7 V to -0.1 V *vs.* RHE.

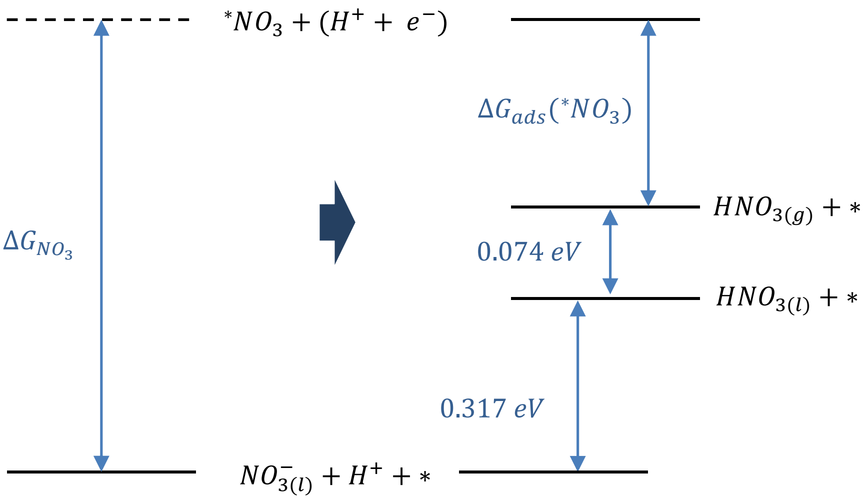

Supplementary Fig. S34. The thermodynamic cycle used to calculate the Gibbs free energy of NO_3^- in the aqueous phase ($NO^-_{3(l)}$) adsorbed onto the electrode surface[2,3]. The thermodynamic values (0.317 eV and 0.074 eV) are obtained from the CRC handbook of chemistry and physics[4].

Supplementary Tables

Supplementary Table S1. Summary of ICP-OES analysis results for different Cu/Co ratio.

	Cu:Co(%)
	ICP
$Cu_{65}Co_{35}$	66:34
$Cu_{50}Co_{50}$	51:49
$Cu_{15}Co_{85}$	14:86

Supplementary Table S2. Comparison of the electrocatalytic NO_3^-RR performances of $Cu_{50}Co_{50}$ catalysts with other extensively reported electrocatalysts.

Cathode Material	Maximum FE for NH_3	Bias-Current Density for NH_3	Maximum NH_3 Production Reported	Conditions	Potential	Ref.
**PA-RhCu cNCs**	93.7%	-	0.254 mg cm^{-2} h^{-1}, 2.40 mg mg_{cat}^{-1} h^{-1}	50 mM KNO_3, 0.1 M $HClO_4$	0.05V *vs.* RHE	5
**oxo-MoS_x**	96%	-	-	1006 mM NO_3^-, 1 mM PBS	0V *vs.* RHE	6
**$Cu_{50}Ni_{50}$**	99%	-90 mA cm^{-2}	-	100 mM NO_3^-, 1 M KOH	-0.1V *vs.* RHE	1
**Co-NAs**	100.0%	-	4.16 mmol cm^{-2} h^{-1}, 2.6 mol g_{cat}^{-1} h^{-1}	100 mM NO_3^-, 1 M KOH	-0.14 V *vs.* RHE	7
**Cu-NSs**	99.7%	-	390.1 ug_{NH3} cm^{-2} h^{-1}, 390.1 ug_{NH3} mg_{cat}^{-1} h^{-1}	10 mM KNO_3, 0.1 M KOH	-0.15V *vs.* RHE	8
**CuCoSP**	93.3%	-300 mA cm^{-2}	1.17 mol cm^{-2} h^{-1}	100 mM NO_3^-, 0.1 M KOH	-0.175 V *vs.* RHE	9
**$Ni_3B@NiB_{2.74}$**	~ 100%	-25mA cm^{-2}	0.1983 mmol cm^{-2} h^{-1}	100 mM KNO_3, 0.1 M KOH	-0.2V *vs.* RHE	10
**Strained Ru-NCs**	~ 100%	-120 mA cm^{-2}	1.17 mmol cm^{-2} h^{-1}, 5.56 mol g_{cat}^{-1} h^{-1}	1 M NO_3^-, 1 M KOH	-0.2V *vs.* RHE	11
**Cu/Cu_2O NWAs**	95.8%	-	0.2449 mmol cm^{-2} h^{-1}	14.3 mM NO_3^-, 0.5 M Na_2SO_4	-0.85V *vs.* SCE	12
**Rh@Cu**	93.0%	-162 mA cm^{-2}	1.27 mmol cm^{-2} h^{-1}	100 mM NO_3^-, 0.1 M Na_2SO_4	-0.2 V *vs.* RHE	13
**FOSP-Cu**	93.9%	-	101.4 μmol cm^{-2} h^{-1}	0.1 M KNO3, 0.5 M Na2SO4	-0.266 V *vs.* RHE	14
**CoO_x**	93.40%	-	0.1723 mmol cm^{-2} h^{-1}, 82.4 ± 4.8 mg mg_{cat}^{-1} h^{-1}	100 mM KNO_3, 0.1 M KOH	-0.3 V *vs.* RHE	15
**a-RuO_2**	97.5%	-	0.1158 mmol cm^{-2} h^{-1}	200 ppm nitrate-N. 0.5 M Na_2SO_4	-0.35 V *vs.* RHE	16
**Cu-PTCDA**	85.9%	-	0.0256 mmol cm^{-2} h^{-1}	36 mM NO_3^-, 0.1 mM PBS	-0.4V *vs.* RHE	17
**TiH_2/Ti**	74.7%	-	-	10 mM HNO_3, 1 M $NaClO_4$	-0.8 V *vs.* RHE	18
**TiH_2/Ti**	38.0%	-	-	10 mM HNO_3, 1 M $NaClO_4$	-0.4 V *vs.* RHE	18
**Fe/Ni_2P**	94.30%	-	4.17 mg_{NH3} cm^{-2} h^{-1}	500 mM KNO_3, 0.1 M K_2SO_4	-0.4 V *vs.* RHE	19
**Pd Octohedron**	79.9%	-	0.5485 mmol cm^{-2} h^{-1}, 2.74 mmol mg_{cat}^{-1} h^{-1}	0.1 M NO_3^-, 0.1 M Na_2SO_4	-0.7 V *vs.* RHE	20
**Fe single atom**	75.0%	-100 mA cm^{-2}	0.46 mmol cm^{-2} h^{-1}, 20 mmol mg_{cat}^{-1} h^{-1}	500 mM KNO_3, 0.1 M K_2SO_4	-0.85 V *vs.* RHE	21
**TiO_{2-x}**	85%	-	0.045 mmol cm^{-2} h^{-1}, 0.045 mmol mg_{cat}^{-1} h^{-1}	3.6 mM NO_3^-, 0.5 M Na_2SO_4	-1.6V *vs.* SCE	22
**TiO_2**	66.3%	-	0.024 mmol cm^{-2} h^{-1}, 0.024 mmol mg_{cat}^{-1} h^{-1}	3.6 mM NO_3^-, 0.5 M Na_2SO_4	-1.6V *vs.* SCE	22
**Ti**	82%	-22 mA cm^{-2}	-	0.3 mM KNO_3, 0.1 M HNO_3	-1V *vs.* RHE	23
**Pd-NDs/Zr-MOF**	58.10%	-	0.1149 mmol cm^{-2} h^{-1}, 287.31 mmol g_{cat}^{-1} h^{-1}	500 ppm NO_3^-, 0.1 M Na_2SO_4	-1.3 V *vs.* RHE	24
**$Cu_{50}Co_{50}$**	**51 ± 2 %**	**-6 mA cm^{-2}**	**0.029 mmol cm^{-2} h^{-1}, 5.9 mmol g_{cat}^{-1} h^{-2}**	**100 mM NO_3^-, 1 M KOH**	**0.4 V *vs.* RHE**	**This work**
	**46 ± 5%**	**-8 mA cm^{-2}**	**0.031 mmol cm^{-2} h^{-1}, 6.2 mmol g_{cat}^{-1} h^{-1}**	**100 mM NO_3^-, 1 M KOH**	**0.3 V *vs.* RHE**	
	**41 ± 1 %**	**-9 mA cm^{-2}**	**0.026 mmol cm^{-2} h^{-1}, 5.2 mmol g_{cat}^{-1} h^{-0}**	**100 mM NO_3^-, 1 M KOH**	**0.2 V *vs.* RHE**	
	**65 ± 2 %**	**-26 mA cm^{-2}**	**0.08 mmol cm^{-2} h^{-1}, 16 mmol g_{cat}^{-1} h^{-2}**	**100 mM NO_3^-, 1 M KOH**	**0.1 V *vs.* RHE**	

	**88 ± 2%**	**-395 mA cm^{-2}**	**1.62 mmol cm^{-2} h^{-1}, 324 mmol g$_{cat}$$^{-1}$ h^{-1}**	**100 mM NO_3^-, 1 M KOH**	**0.0 V *vs.* RHE**
	**97 ± 5 %**	**-942 mA cm^{-2}**	**4.27 mmol cm^{-2} h^{-1}, 854 mmol g$_{cat}$$^{-1}$ h^{-0}**	**100 mM NO_3^-, 1 M KOH**	**-0.1 V *vs.* RHE**
	**100 ± 1 %**	**-1035 mA cm^{-2}**	**4.83 mmol cm^{-2} h^{-1}, 960 mmol g$_{cat}$$^{-1}$ h^{-1}**	**100 mM NO_3^-, 1 M KOH**	**-0.2 V *vs.* RHE**
	**98 ± 3 %**	**-1064 mA cm^{-2}**	**4.87 mmol cm^{-2} h^{-1}, 974 mmol g$_{cat}$$^{-1}$ h^{-2}**	**100 mM NO_3^-, 1 M KOH**	**-0.3 V *vs.* RHE**
	**90 ± 6 %**	**-1098 mA cm^{-2}**	**4.58 mmol cm^{-2} h^{-1}, 916 mmol g$_{cat}$$^{-1}$ h^{-3}**	**100 mM NO_3^-, 1 M KOH**	**-0.4 V *vs.* RHE**

Supplementary Table S3. FTIR peaks for the *in situ* NO_3^-RR on $Cu_{50}Co_{50}$, Cu and Co catalysts.

**Wavenumber / cm^{-1}**				**reference**
**$Cu_{50}Co_{50}$**	**Cu**	**Co**	**Assignment**	
1110	1123	1110	ν_s(N-O) of NH_2OH	25,26
1236	1235	-	ν_a(N-O) of NO_2^-	25
1354	1354	1353	ν_a(N-O) of NO_3^-	27
1392	1392	1393	ν_s(N-O) of NO_3^-	27
1557	1530	1580	ν(N-O) of bridge bonded NO_{ad}	27,28
1639	1656	1655	ν(N-O) of on-top bonded NO_{ad}	27,28
1638	1638	1633	δ(HOH) of H_2O	29
2109-2085	-	2115-2092	ν(Co-H)	30,31

Supplementary Table S4. Raman scattering peaks for *in situ* nitrates reduction on $Cu_{50}Co_{50}$, Cu and Co catalysts.

**Ramam shift / cm^{-1}**				**reference**
**$Cu_{50}Co_{50}$**	**Cu**	**Co**	**Assignment**	
431	427	-	Cu-O_x	32
568	-	568	Co-O_x	33
625	625	-	Cu_2O	34
714	714	-	Cu-OH_{ad}	32,35
-	800	-	NO_2^- bending	36
998	996	996	ν(NO) of unidentate nitrato	37
1028	1027	1029	$\nu_s(NO_3^-)$ adsorbed NO_3^-	38
1049	1049	1051	$\nu_s(NO_3^-)$	39
1125	1127	1127	ν_s NO_2^-, chelating nitrito	40
1254	1242	1244	ν_a NO_2^-, chelating nitrito	40
1315	1307	1312	δ_s(HNH) deformation	38,41
1374	1378	1381	δ_s(HNH) deformation	38,41
1439	1436	1441	ν(N=O) ,bridging nitro	41
1540	1537	1545	ν(N=O) of HNO	42
1591	1564	1599	δ_a(HNH) of NH3	43

Supplementary Table S5. The electronic energies (E), zero-point energies (ZPE) and entropy (TS) corrections for $H_2O_{(l)}$, $H_{2(g)}$, $NH_{3(g)}$ and $HNO_{3(g)}$ used in the Gibbs free energy calculations. The data marked in bold are cited from CRC handbook of chemistry and physics.[4] (T = 298.15 K, p_{gas} = 1 atm).

Molecule	E / eV	ZPE / eV	TS / eV	TS(CRC handbook) / eV
$H_2O_{(l)}$	-14.22	0.57	0.67	\
$H_{2(g)}$	-6.77	0.27	0.40	**0.40**
$NH_{3(g)}$	-19.54	0.91	0.60	**0.60**
$HNO_{3(g)}$	-28.61	0.69	0.83	**0.82**

Supplementary Table S6. Adsorption configurations of $*NO_3$, $*NO_2$, NO, $*NOH$, $*NHOH$, $*NH_2OH$, $*NH_2$ and $*NH_3$ on Cu(111), CuCo(111), Co(111) surfaces.

	Cu	CuCo	Co
$*NO_3$	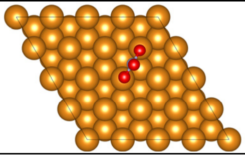	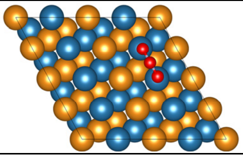	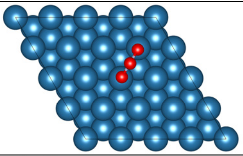
$*NO_2$	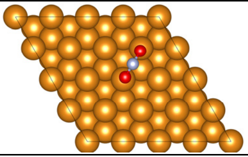	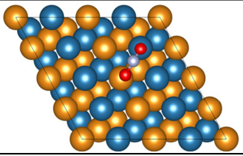	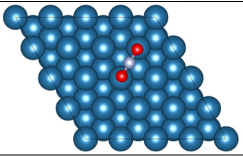
*NO	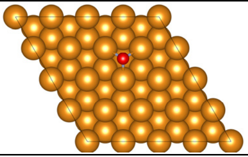	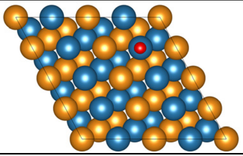	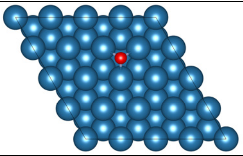
*NOH	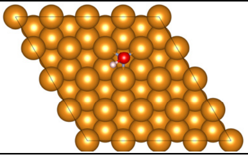	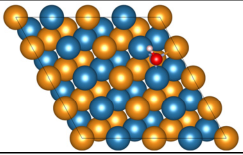	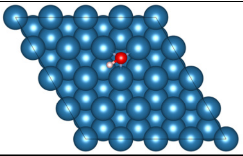
*NHOH	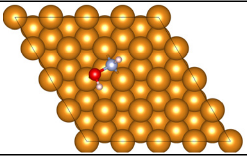	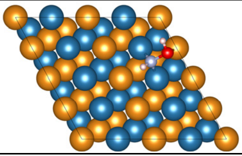	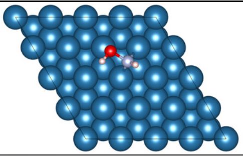
$*NH_2OH$	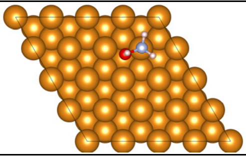	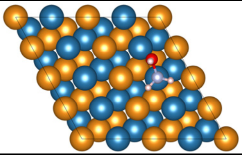	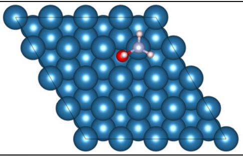
$*NH_2$	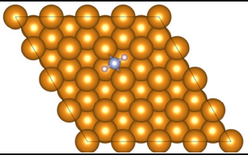	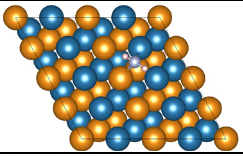	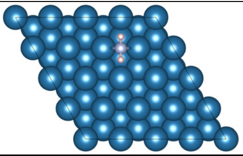
$*NH_3$	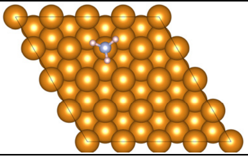	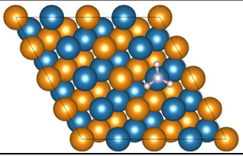	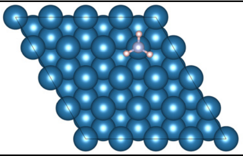

Supplementary Table S7. The correction of zero-point energy (ZPE) of adsorption species on different catalysts' surfaces (in eV). All the slab atoms were fixed during the vibrational analyses.

	Cu(111)	Co(111)	CuCo(111)
*NO_3	0.39	0.40	0.40
*NO_2	0.26	0.27	0.26
*NO	0.16	0.17	0.19
*NOH	0.46	0.45	0.46
*NHOH	0.78	0.77	0.77
*NH_2OH	1.10	1.11	1.10
*NH_2	0.67	0.68	0.67
*NH_3	0.99	1.01	1.01
*H	0.17	0.17	0.18

Supplementary Table S8. The correction of entropy (TS) of adsorption species on different catalysts' surfaces (in eV). All the slab atoms were fixed during the vibrational analyses. (T = 298.15 K)

	Cu(111)	Co(111)	CuCo(111)
*NO_3	0.23	0.27	0.26
*NO_2	0.24	0.20	0.22
*NO	0.14	0.12	0.17
*NOH	0.16	0.16	0.15
*NHOH	0.15	0.14	0.15
*NH_2OH	0.21	0.19	0.25
*NH_2	0.10	0.09	0.09
*NH_3	0.14	0.17	0.12
*H	0.00	0.00	0.00

Supplementary Table S9. Calculated electronic energies (E) of adsorption species on different catalysts' surfaces (in eV).

	Cu(111)	Co(111)	CuCo(111)
*	-162.86	-317.95	-233.09
$*NO_3$	-188.29	-343.62	-258.94
$*NO_2$	-182.66	-338.01	-252.94
*NO	-176.57	-332.66	-247.60
*NOH	-179.97	-335.96	-251.05
*NHOH	-183.84	-339.45	-254.66
$*NH_2OH$	-187.60	-342.90	-258.09
$*NH_2$	-178.82	-334.23	-249.18
$*NH_3$	-182.92	-338.19	-253.36
*H	-166.57	-321.82	-236.97

Supplementary Table S10. Calculated Gibbs free energies (ΔG) of adsorption species for NO_3^-RR on different catalysts' surfaces with respect to the reference $NO_3^-{}_{(l)}$ + * (in eV).

	Cu(111)		Co(111)		CuCo(111)	
	U = 0 V	U = -0.2 V	U = 0 V	U = -0.2 V	U = 0 V	U = -0.2 V
Reference $NO_3^-{}_{(l)}$ + *	0	0	0	0	0	0
$\Delta G(*NO_3)$	0.41	0.61	0.14	0.34	-0.03	0.17
$\Delta G(*NO_2)$	-1.50	-1.70	-1.72	-1.92	-1.54	-1.74
ΔG(*NO)	-2.83	-3.43	-3.80	-4.40	-3.63	-4.23
ΔG(*NOH)	-2.49	-3.29	-3.41	-4.21	-3.33	-4.13
ΔG(*NHOH)	-2.58	-3.58	-3.10	-4.10	-3.18	-4.18
$\Delta G(*NH_2OH)$	-2.63	-3.83	-2.81	-4.01	-2.93	-4.13
$\Delta G(*NH_2)$	-5.03	-6.43	-5.34	-6.74	-5.16	-6.56
$\Delta G(*NH_3)$	-5.39	-6.99	-5.60	-7.20	-5.58	-7.18
$\Delta G(NH_3)$	-5.42	-7.02	-5.42	-7.02	-5.42	-7.02

Supplementary Table S11. Calculated Gibbs free energies (ΔG) of adsorption species for HER on different catalysts' surfaces with respect to the reference of $H^+ + e^- + *$ (in eV).

	Cu(111)		Co(111)		CuCo(111)	
	U = 0 V	U = -0.2 V	U = 0 V	U = -0.2 V	U = 0 V	U = -0.2 V
Reference $H^+ + e^- + *$	0	0	0	0	0	0
ΔG(*H)	-0.08	-0.28	-0.25	-0.45	-0.25	-0.45

Supplementary Table S12. top view and side view of Cu(111), Co(111) and CuCo(111) with different surface exposure structures and the corresponding adsorption configuration of $*NO_3$ and $*NO_2$

		Cu	CuCo	CuCo-1	CuCo-2	CuCo-3	CuCo-4	CuCo-5	Co
slab	top view	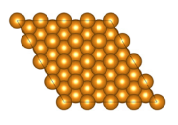	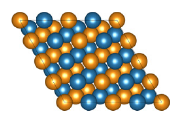	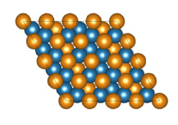	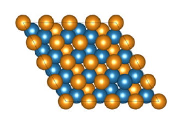	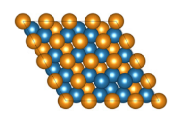	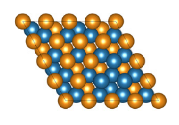	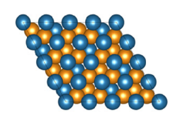	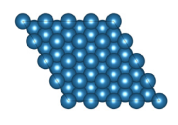
	side view	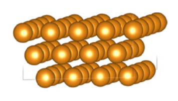	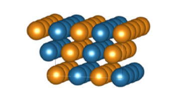	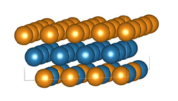	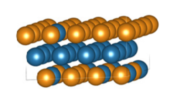	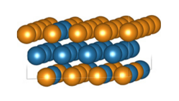	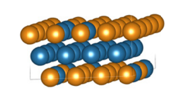	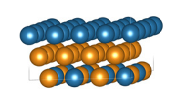	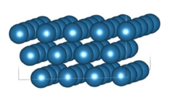
$*NO_3$		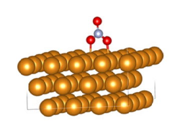	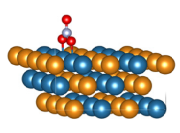	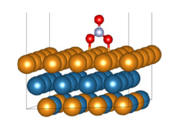	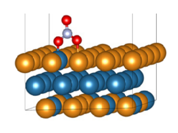	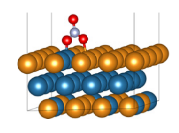	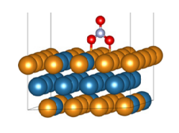	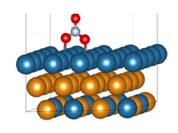	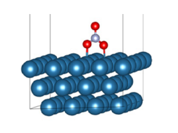
$*NO_2$		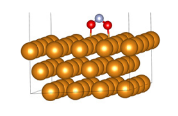	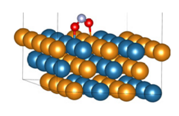	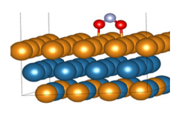	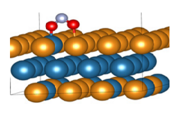	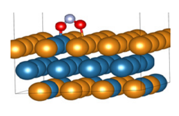	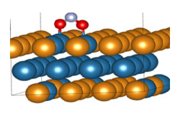	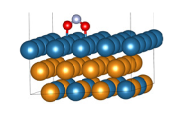	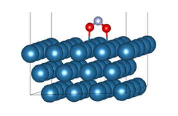

Supplementary Table S13. Calculated Gibbs free energies (in eV) of $*NO_3$ and $*NO_2$ on Cu(111), Co(111) and CuCo(111) with different surface exposure structures at 0 V *vs.* RHE.

	Cu	CuCo	CuCo-1	CuCo-2	CuCo-3	CuCo-4	CuCo-5	Co
Reference $NO_3^-{}_{(l)}$ + *	0	0	0	0	0	0	0	0
$\Delta G(*NO_3)$	0.41	-0.03	0.33	0.30	0.30	0.10	0.22	0.14
$\Delta G(*NO_2)$	-1.50	-1.54	-1.41	-1.53	-1.57	-1.76	-1.66	-1.72

Supplementary Table S14. Calculated Gibbs free energies (in eV) of $*NO_3$ and $*NO_2$ on Cu(111), Co(111) and CuCo(111) with different surface exposure structures at -0.2 V *vs.* RHE.

	Cu	CuCo	CuCo-1	CuCo-2	CuCo-3	CuCo-4	CuCo-5	Co
Reference $NO_3^-{}_{(l)}$ + *	0	0	0	0	0	0	0	0
$\Delta G(*NO_3)$	0.61	0.17	0.53	0.50	0.50	0.30	0.42	0.34
$\Delta G(*NO_2)$	-1.70	-1.74	-1.61	-1.73	-1.77	-1.96	-1.86	-1.92

Supplementary Notes.

Supplementary Note S1. Details of DFT calculations.

1. The Gibbs free energy of $NO^-_{3(l)}$ adsorption onto the electrode surface.

At 0 V vs. RHE, $H^+ + e^- \rightleftharpoons \frac{1}{2}H_2$ is in equilibrium at $p_{H_2} = 1$ atm (Eq. (S1) and (S2)).

$$G(H^+) + G(e^-) = \frac{1}{2}G(H_2) \qquad (S1)$$

$$G(OH^-) = G(H_2O) - \frac{1}{2}G(H_2) \qquad (S2)$$

The process of $NO^-_{3(l)}$ adsorbed onto the electrode surface (Eq. (S3)) was divided into three steps (Eqs. (S4-S6)), as shown in **Supplementary Fig. S34**[2,3].

Firstly, the Gibbs free energy for $HNO_{3(l)}$ formation from $NO^-_{3(l)}$ is 0.317 eV (Eq. (S4)). The change in Gibbs free energy of the vaporization of $HNO_{3(l)}$ was then calculated from the Gibbs free energy difference between the standard formation of HNO_3 in liquid ($HNO_{3(l)}$) (-0.836 eV) and gas phase ($HNO_{3(g)}$) (-0.762 eV) and was equal to 0.074 eV (Eq. (S5)). The Gibbs free energy of NO_3 adsorption on the surface ($^*NO_3$) (Eq.(S6)), following the Eq. (S7).

$$NO^-_{3(l)} + * \longrightarrow {}^*NO_3 + e^- \qquad (S3)$$

$$NO^-_{3(l)} + H^+ \longrightarrow HNO_{3(l)} \qquad (\Delta G = 0.317 \text{ eV}) \qquad (S4)$$

$$HNO_{3(l)} \longrightarrow HNO_{3(g)} \qquad (\Delta G = 0.074 \text{ eV}) \qquad (S5)$$

$$HNO_{3(g)} + * \longrightarrow {}^*NO_3 + \frac{1}{2}H_{2(g)} \qquad (S6)$$

$$\Delta G_{ads}(^*NO_3) = G(^*NO_3) + \frac{1}{2}G_{gas}(H_2) - G(*) - G_{gas}(HNO_3) \qquad (S7)$$

Ultimately, the overall Gibbs free energy change (ΔG_{*NO_3}) for $NO_3^-{}_{(l)}$ adsorption from the solution phase on to electrode surface ($NO_3^-{}_{(l)} + * \rightarrow {}^*NO_3 + e^-$) was calculated following Eq. (S8):

$$\Delta G_{*NO_3} = \Delta G_{ads}(^*NO_3) + 0.074 \text{ eV} + 0.317 \text{ eV} - eU$$

$$= G(^*NO_3) + \frac{1}{2}G_{gas}(H_2) - G(*) - G_{gas}(HNO_3) + 0.391 \text{ eV} - eU$$

$$= E(^*NO_3) + ZPE(^*NO_3) - TS(^*NO_3) + \frac{1}{2}[(E_{gas}(H_2) + ZPE_{gas}(H_2) - TS_{gas}(H_2)] -$$

$$E(*) - E_{gas}(HNO_3) - ZPE_{gas}(HNO_3) + TS_{gas}(HNO_3) + 0.391 \text{ eV} - eU \qquad (S8)$$

Here we neglected the ZPE and rotational, translational, and vibrational free energy contributions for slab (i.e., G(*) = E(*)).[2] The U is the potential at the electrode and e is the transferred charge.

2. The NO_3^-RR on different catalysts surfaces were simulated according to the following reactions[44]:

$$NO^-_{3(l)} + * \rightarrow * NO_3 + e^- \qquad (S9)$$

$$* NO_3 + H_2O + 2e^- \rightarrow * NO_2 + 2OH^- \qquad (S10)$$

$$* NO_2 + H_2O + 2e^- \rightarrow * NO + 2OH^- \qquad (S11)$$

$$* NO + H_2O + e^- \rightarrow * NOH + OH^- \qquad (S12)$$

$$* NOH + H_2O + e^- \rightarrow * NHOH + OH^- \qquad (S13)$$

$$* NHOH + H_2O + e^- \rightarrow * NH_2OH + OH^- \qquad (S14)$$

$$* NH_2OH + e^- \rightarrow * NH_2 + OH^- \qquad (S15)$$

$$* NH_2 + H_2O + e^- \rightarrow * NH_3 + OH^- \qquad (S16)$$

$$* NH_3 \rightarrow * + NH_3 \quad (S17)$$

Where the * represents the active sites.

For each subsequent reaction, the free energies were given after gas correction, following Eq. (S18):

$$\Delta G = \Delta E + \Delta ZPE - T\Delta S - eU \quad (S18)$$

where ΔE is the energy obtained by the difference between reactant and product, ΔZPE denotes the change of zero-point energy. ΔS is the change in entropy for each reaction. The entropies of adsorbate and adsorption site are negligible. Here, U is the potential at the electrode and e is the transferred charge.

Supplementary Note S2. The reference used in the Gibbs free energy calculations. In the thermodynamic calculation, the energy of negatively charged species like NO_3^- and OH^- are difficult to determine. However, they can be approached using the stable molecules HNO_3 and H_2 in the gas phase ($HNO_{3(g)}$ and $H_{2(g)}$) and liquid H_2O ($H_2O_{(l)}$) instead. The $HNO_{3(g)}$, $H_2O_{(l)}$, $H_{2(g)}$ and a clean slab are used as references when calculating the Gibbs free energies of reaction intermediates. The zero-point energies (ZPE) and entropy (TS) (in eV) corrections for Gibbs free energy calculations of $HNO_{3(g)}$, $H_2O_{(l)}$, $NH_{3(g)}$ and $H_{2(g)}$ were obtained through vibrational analyses. The calculated TS were close to the data cited from CRC handbook of chemistry and physics[4], as shown in **Supplementary Table S5**.

Supplementary Note S3. The initial date calculated for the Gibbs free energy calculations. The correction of zero-point energy (ZPE), entropy (TS) and calculated electronic energies (E) of the intermediates involved in NO_3^-RR on Cu(111), Co(111) and CuCo(111) surfaces were listed in the **Supplementary Table S7**, **Supplementary Table S8** and **Supplementary Table S9**, respectively. All the slab atoms were fixed during the vibrational analyses. (T = 298.15 K)

Supplementary Note S4. Stable configurations of the intermediates on different CuCo surface models. To study the adsorption mode of intermediate species and active sites, we studied the process of $* + NO^-_{3(l)} \rightarrow *NO_3 \rightarrow *NO_2$ on the 6 different CuCo modes, since $* + NO^-_{3(l)} \rightarrow *NO_3$ is the rate-determining step. For the reason of computing resources limitation, the following processes were omitted. The models were shown below (**Supplementary Table S12**). The Gibbs free energies of $*NO_3$ and $*NO_2$ on Cu(111), Co(111) and CuCo(111) with a different arrangement of surface atoms, denoted as CuCo and CuCo1 to 5, were calculated and presented in **Supplementary Table S13 and S14**. The atom's arrangement on the first layer of the alloy directly affects the adsorption of intermediates. We first compared the ΔG ($*NO_3$) of Cu and CuCo-1, which had the same surface composition and active sites for adsorption. The ΔG ($*NO_3$) on CuCo-1 was slightly lower than that on Cu, indicating that the alloying between Cu and Co benefits the NO_3^-RR. As the appearance and gradually increase of Co atoms ratio in the first layer (CuCo-2, 3, 4 and CuCo), the ΔG($*NO_3$) required for NO_3^- adsorption gradually decreased from 0.53 eV to 0.17 eV in the case of -0.2 V and from 0.33 eV to -0.03 eV in the case of 0.0 V. It demonstrated that the surface layer element composition significantly affected the adsorption mode. However, it should be noted

that when the surface layer composes only Co atoms, the adsorption energy of NO_3^- will increase. The trend was consistent with the experimental result that $\eta_{10\ mA\ cm^{-2}}$ of NO_3^-RR on Co catalyst was 690 mV that is more negative than on $Cu_{50}Co_{50}$ catalyst ($\eta_{10\ mA\ cm^{-2}}$ of 498 mV) (**Fig. 2a** in the manuscript). It should be mentioned that a dramatically higher j_{NH_3} was obtained on $Cu_{50}Co_{50}$ catalyst at 0.0 V (347 mA cm^{-2}) compared to Cu catalyst (34 mA cm^{-2}) and Co catalyst (21 mA cm^{-2}) alone (S**upplementary Fig. S24a**). The CuCo model provided more consistent results of DFT calculation with the above experimental results. Therefore, we selected the CuCo model for subsequent analysis to explain the difference between the $Cu_{50}Co_{50}$ alloy and the pure Cu and Co metal catalysts.

References for Supplementary Information.

1 Wang, Y. *et al.* Enhanced Nitrate-to-Ammonia Activity on Copper-Nickel Alloys via Tuning of Intermediate Adsorption. *J. Am. Chem. Soc.* **142**, 5702-5708 (2020).

2 Liu, J.-X., Richards, D., Singh, N. & Goldsmith, B. R. Activity and Selectivity Trends in Electrocatalytic Nitrate Reduction on Transition Metals. *ACS Catal.* **9**, 7052-7064 (2019).

3 Calle-Vallejo, F., Huang, M., Henry, J., Koper, M. T. M. & Bandarenka, A. S. Theoretical design and experimental implementation of Ag/Au electrodes for the electrochemical reduction of nitrate. *Physical chemistry chemical physics : PCCP* **15 9**, 3196-3202 (2013).

4 Lide, D. R. *CRC Handbook of Chemistry and Physics*. (CRC Press, Boca Raton, FL, 2009).

5 Ge, Z. X. *et al.* Interfacial Engineering Enhances the Electroactivity of Frame-Like Concave RhCu Bimetallic Nanocubes for Nitrate Reduction. *Adv. Energy Mater.* (2022).

6 Li, Y. *et al.* Enzyme Mimetic Active Intermediates for Nitrate Reduction in Neutral Aqueous Media. *Angew. Chem. Int. Ed.* **59**, 9744-9750 (2020).

7 Deng, X., Yang, Y., Wang, L., Fu, X. Z. & Luo, J. L. Metallic Co Nanoarray Catalyzes Selective NH_3 Production from Electrochemical Nitrate Reduction at Current Densities Exceeding 2 A cm^{-2}. *Adv. Sci.* **8**, 2004523 (2021).

8 Fu, X. *et al.* Alternative route for electrochemical ammonia synthesis by reduction of nitrate on copper nanosheets. *Appl. Mater. Today* **19** (2020).

9 He, W. *et al.* Splicing the active phases of copper/cobalt-based catalysts achieves high-rate tandem electroreduction of nitrate to ammonia. *Nat. Commun.* **13**, 1129 (2022).

10 Li, L. *et al.* Efficient Nitrogen Fixation to Ammonia through Integration of Plasma Oxidation with Electrocatalytic Reduction. *Angew. Chem. Int. Ed.* **60**, 14131-14137 (2021).

11 Li, J. *et al.* Efficient Ammonia Electrosynthesis from Nitrate on Strained Ruthenium Nanoclusters. *J. Am. Chem. Soc.* **142**, 7036-7046 (2020).

12 Wang, Y., Zhou, W., Jia, R., Yu, Y. & Zhang, B. Unveiling the Activity Origin of a Copper-based Electrocatalyst for Selective Nitrate Reduction to Ammonia. *Angew. Chem. Int. Ed.* **59**, 5350-5354 (2020).

13 Liu, H. *et al.* Efficient Electrochemical Nitrate Reduction to Ammonia with Copper-Supported Rhodium Cluster and Single-Atom Catalysts. *Angew. Chem. Int. Ed.* **61**, e202202556 (2022).

14 Zhao, Y. *et al.* Flower-like open-structured polycrystalline copper with synergistic multi-crystal plane for efficient electrocatalytic reduction of nitrate to ammonia. *Nano Energy* **97** (2022).

15 Wang, J. *et al.* Electrocatalytic Reduction of Nitrate to Ammonia on Low-Cost Ultrathin CoO_x Nanosheets. *ACS Catal.* **11**, 15135-15140 (2021).

16 Wang, Y. *et al.* Structurally Disordered RuO_2 Nanosheets with Rich Oxygen Vacancies for Enhanced Nitrate Electroreduction to Ammonia. *Angew. Chem. Int. Ed.* **61**, e202202604 (2022).

17 Chen, G.-F. *et al.* Electrochemical reduction of nitrate to ammonia via direct eight-electron transfer using a copper–molecular solid catalyst. *Nat. Energy* **5**, 605-613 (2020).

18 Liu, M. J. *et al.* Catalytic Performance and Near-Surface X-ray Characterization of Titanium Hydride Electrodes for the Electrochemical Nitrate Reduction Reaction. *J. Am. Chem. Soc.* **144**, 5739-5744 (2022).

19 Zhang, R. *et al.* Efficient Ammonia Electrosynthesis and Energy Conversion through a Zn-Nitrate Battery by Iron Doping Engineered Nickel Phosphide Catalyst. *Adv. Energy Mater.* **12**

(2022).

20 Han, Y. *et al.* Facet-controlled palladium nanocrystalline for enhanced nitrate reduction towards ammonia. *J. Colloid Interface Sci.* **600**, 620-628 (2021).

21 Wu, Z. Y. *et al.* Electrochemical ammonia synthesis via nitrate reduction on Fe single atom catalyst. *Nat. Commun.* **12**, 2870 (2021).

22 Jia, R. *et al.* Boosting Selective Nitrate Electroreduction to Ammonium by Constructing Oxygen Vacancies in TiO_2. *ACS Catal.* **10**, 3533-3540 (2020).

23 McEnaney, J. M. *et al.* Electrolyte Engineering for Efficient Electrochemical Nitrate Reduction to Ammonia on a Titanium Electrode. *ACS Sustain. Chem. Eng.* **8**, 2672-2681 (2020).

24 Jiang, M. *et al.* Interfacial Reduction Nucleation of Noble Metal Nanodots on Redox-Active Metal-Organic Frameworks for High-Efficiency Electrocatalytic Conversion of Nitrate to Ammonia. *Nano Lett.* **22**, 2529-2537 (2022).

25 Pérez-Gallent, E., Figueiredo, M. C., Katsounaros, I. & Koper, M. T. M. Electrocatalytic reduction of Nitrate on Copper single crystals in acidic and alkaline solutions. *Electrochim. Acta* **227**, 77-84 (2017).

26 Rosca, V., Beltramo, G. L. & Koper, M. T. M. Hydroxylamine electrochemistry at polycrystalline platinum in acidic media: a voltammetric, DEMS and FTIR study. *J. Electroanal. Chem.* **566**, 53-62 (2004).

27 Figueiredo, M. C., Souza-Garcia, J., Climent, V. & Feliu, J. M. Nitrate reduction on Pt(111) surfaces modified by Bi adatoms. *Electrochem. commun.* **11**, 1760-1763 (2009).

28 Agrawal, V. K. & Trenary, M. An infrared study of NO adsorption at defect sites on Pt(111). *Surf. Sci.* **259**, 116-128 (1991).

29 Ataka, K., Yotsuyanagi, Takao & Osawa, M. Potential-Dependent Reorientation of Water Molecules at an Electrode/Electrolyte Interface Studied by Surface-Enhanced Infrared Absorption Spectroscopy. *J. Chem. Phys.* **100**, 10664-10672 (1996).

30 Yao, Y., Zhu, S., Wang, H., Li, H. & Shao, M. A Spectroscopic Study on the Nitrogen Electrochemical Reduction Reaction on Gold and Platinum Surfaces. *J. Am. Chem. Soc.* **140**, 1496-1501 (2018).

31 Zhu, S. *et al.* The role of ruthenium in improving the kinetics of hydrogen oxidation and evolution reactions of platinum. *Nat. Catal.* **4**, 711-718 (2021).

32 Zhao, Y. *et al.* Speciation of Cu Surfaces During the Electrochemical CO Reduction Reaction. *J. Am. Chem. Soc.* **142**, 9735-9743 (2020).

33 Orlovskaya, N. *et al.* Detection of temperature- and stress-induced modifications of $LaCoO_3$ by micro-Raman spectroscopy. *Phys. Rev. B* **72**, 014122 (2005).

34 Deng, Y., Handoko, A. D., Du, Y., Xi, S. & Yeo, B. S. In Situ Raman Spectroscopy of Copper and Copper Oxide Surfaces during Electrochemical Oxygen Evolution Reaction: Identification of CuIII Oxides as Catalytically Active Species. *ACS Catal.* **6**, 2473-2481 (2016).

35 Bodappa, N. *et al.* Early Stages of Electrochemical Oxidation of Cu(111) and Polycrystalline Cu Surfaces Revealed by in Situ Raman Spectroscopy. *J. Am. Chem. Soc.* **141**, 12192-12196 (2019).

36 Bae, S.-E., Stewart, K. L. & Gewirth, A. A. Nitrate Adsorption and Reduction on Cu(100) in Acidic Solution. *J. Am. Chem. Soc.* **129**, 10171-10180 (2007).

37 Escribano, R. M. *et al.* The low-frequency Raman and IR spectra of nitric acid hydrates. *Vib. Spectrosc.* **43**, 254-259 (2007).

38 Butcher, D. P. & Gewirth, A. A. Nitrate reduction pathways on Cu single crystal surfaces: Effect of oxide and Cl^-. *Nano Energy* **29**, 457-465 (2016).

39 Zhou, Z., Huang, G. G., Kato, T. & Ozaki, Y. Experimental parameters for the SERS of nitrate ion for label-free semi-quantitative detection of proteins and mechanism for proteins to form SERS hot sites: a SERS study. *J. Raman. Spectrosc.* **42**, 1713-1721 (2011).

40 GOODGAME., D. M. L. & HITCHMAN., M. A. Studies of Nitro and Nitrito Complexes. II. Complexes Containing Chelating NO_2 Groups. *Inorg. Chem.* **4**, 721–725 (1964).

41 Nakamoto, K. *Infrared and Raman Spectra of Inorganic and Coordination Compounds*. (John Wiley & Sons, New York, 2008).

42 Ling, Y., Mills, C., Weber, R., Yang, L. & Zhang, Y. NMR, IR/Raman, and structural properties in HNO and RNO (R = alkyl and aryl) metalloporphyrins with implication for the HNO-myoglobin complex. *J. Am. Chem. Soc.* **132**, 1583-1591 (2010).

43 Wen., N. & Brooker., M. H. Ammonium Carbonate, Ammonium Bicarbonate, and Ammonium Carbamate Equilibria: A Raman Study. *J. Phys. Chem.* **99**, 359-368 (1995).

44 Hu, T., Wang, C., Wang, M., Li, C. M. & Guo, C. Theoretical Insights into Superior Nitrate Reduction to Ammonia Performance of Copper Catalysts. *ACS Catal.* **11**, 14417-14427 (2021).
